# Contribution of disordered regions of the essential protein GEMIN5 to RNA binding

**DOI:** 10.26508/lsa.202603860

**Published:** 2026-10-27

**Authors:** Rosario Francisco-Velilla, Azadeh Nahavandi Araghi, Jorge Ramajo, Salvador Abellan, Encarnacion Martinez-Salas

**Affiliations:** 1 Centro de Biología Molecular Severo Ochoa (CBM), CSIC-UAM , Madrid, Spain; 2 Escuela de Doctorado, Universidad Autónoma de Madrid (UAM), Madrid, Spain

## Abstract

GEMIN5 is emerging as a multifunctional RNA-binding protein (RBP) linked to human disease. This RBP comprises a WD40 repeat domain at the N terminus. The C-terminal region (G5C) includes a tetratricopeptide repeat (TPR)-like dimerization module, a non-canonical RNA-binding site (RBS), and an intrinsically disordered region (IDR). Understanding the molecular basis for RNA binding is critical to decipher how GEMIN5 pathological variants impact on their functions. Here we show that the RNA-binding domain consists of an extended binding surface comprising a serine-rich loop and an acidic cluster within the IDR. The RNA binding ability measured in vitro and in soluble cellular extracts indicated that individually, ser-rich motif and acidic cluster mutants diminished RNA binding intensity. However, in the absence of the RBS region, the TPR-extended protein is sufficient to facilitate RNA binding. AlphaFold modeling of the TPR-extended protein shows the assembly of an elongated dimer bound to RNA. The same RBS is observed in the G5C–RNA oligomer model. We propose that the elongated dimer forms an extended binding surface, fine-tuning the RNA binding ability of the G5C architecture.

## Abstract (precis)

The RNA-binding domain of the human GEMIN5 protein consists of an oligomerizing extended surface, including a serine-rich motif and a disordered acidic cluster that cooperatively regulate RNA binding.

## Graphical Abstract

**Figure:**
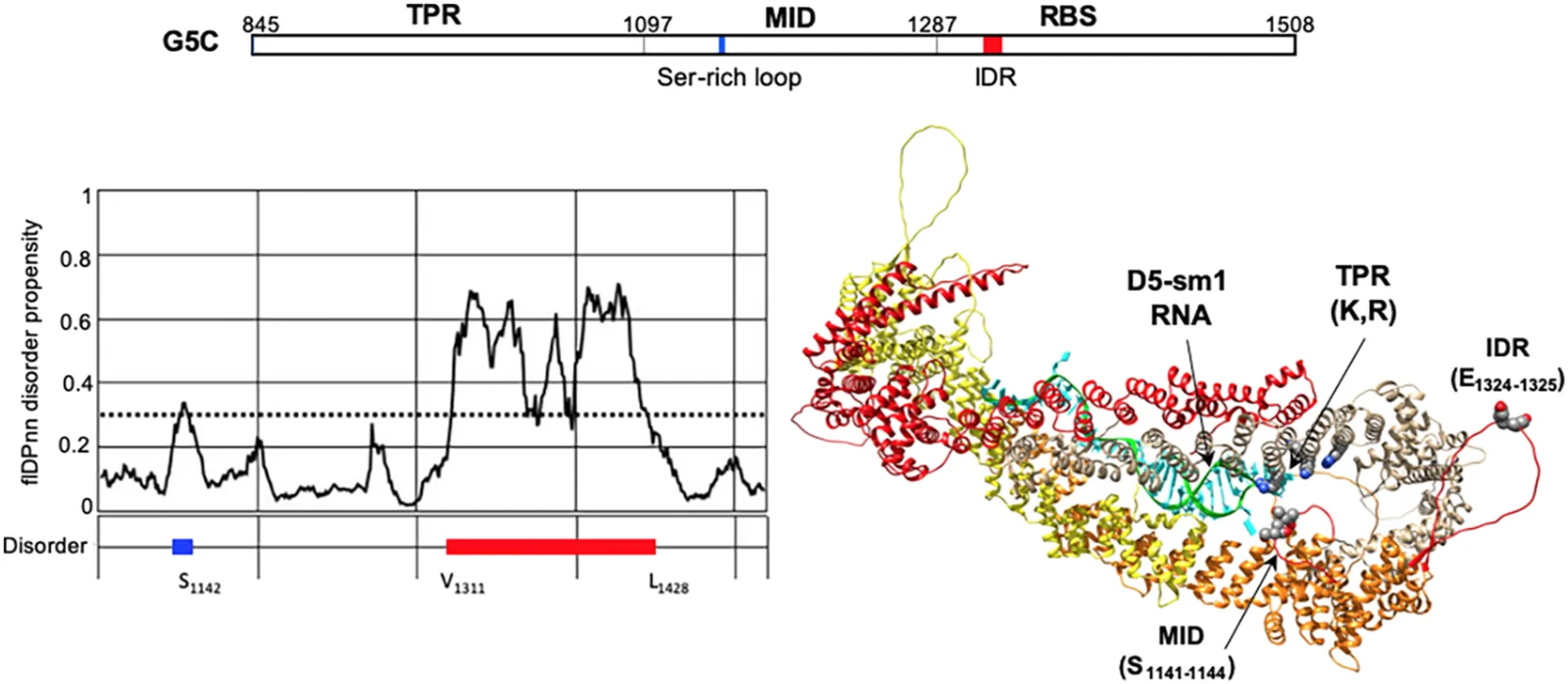

## Introduction

RNA-binding proteins (RBPs) are master regulators of posttranscriptional gene expression. In recent years, the implementation of interactome capture methodologies has discovered a large number of previously unknown RBPs, which contain a wide diversity of canonical and non-canonical RNA-binding domains (RBDs) (1, 2). In turn, RBDs are functionally diverse, such that the particular combination of RBDs in a given protein can confer specific target recognition and, therefore, unique functions (3, 4). Conformational changes due to substitutions of small side-chain amino acids or loop movements in one of the RBDs can affect RNA recognition. Besides, interdomain linkers facilitate protein flexibility, providing enhanced RNA binding via cooperative interactions or induced fit mechanisms. Consequently, the presence of extended sequences between separate domains of RBPs can increase the effective affinities by modifying the geometry of the interacting RNA-binding surface (5).

RBPs frequently harbor intrinsically disordered regions (IDRs), a feature initially thought to facilitate promiscuous interaction with other proteins and nucleic acids (6, 7). Nevertheless, the abundance and conservation of intrinsically disordered RBPs strongly suggest their functional significance (8). Importantly, IDRs in RBPs can alter the recognition of specific targets, at least in part due to modification of the protein binding ability, which in turn facilitates interaction with other proteins (9, 10). In particular, IDRs present in RBPs often contain sequences enriched in aspartate (D), or glutamate (E) (11), regulating RNA binding specificity (12, 13). Negatively charged IDRs can electrostatically mimic nucleic acids and compete with them for positively charged RBDs, leading to a phenomenon known as autoinhibition (14). IDRs can also facilitate proper protein folding (15). Thus, understanding the properties of negatively charged IDRs is important to elucidate its functional role in gene expression.

GEMIN5 is a predominantly cytoplasmic protein, initially identified as the RBP of the survival of motor neurons (SMN) complex (16, 17). The protein contains a WD40 repeat domain at the N terminus (18, 19), and a C-terminal alpha-helical region (G5C), encompassing a tetratricopeptide repeat (TPR)-like dimerization module (20) and a non-canonical RNA-binding site (RBS) (21). A short tract of positively charged and aromatic residues, located at the N terminus of the RBS, determines binding to cognate RNAs (22). In addition, the RBS region of GEMIN5 harbors a conformational flexible IDR region (23). However, the potential role of the IDR coding sequence remains obscure.

**Figure 1.**
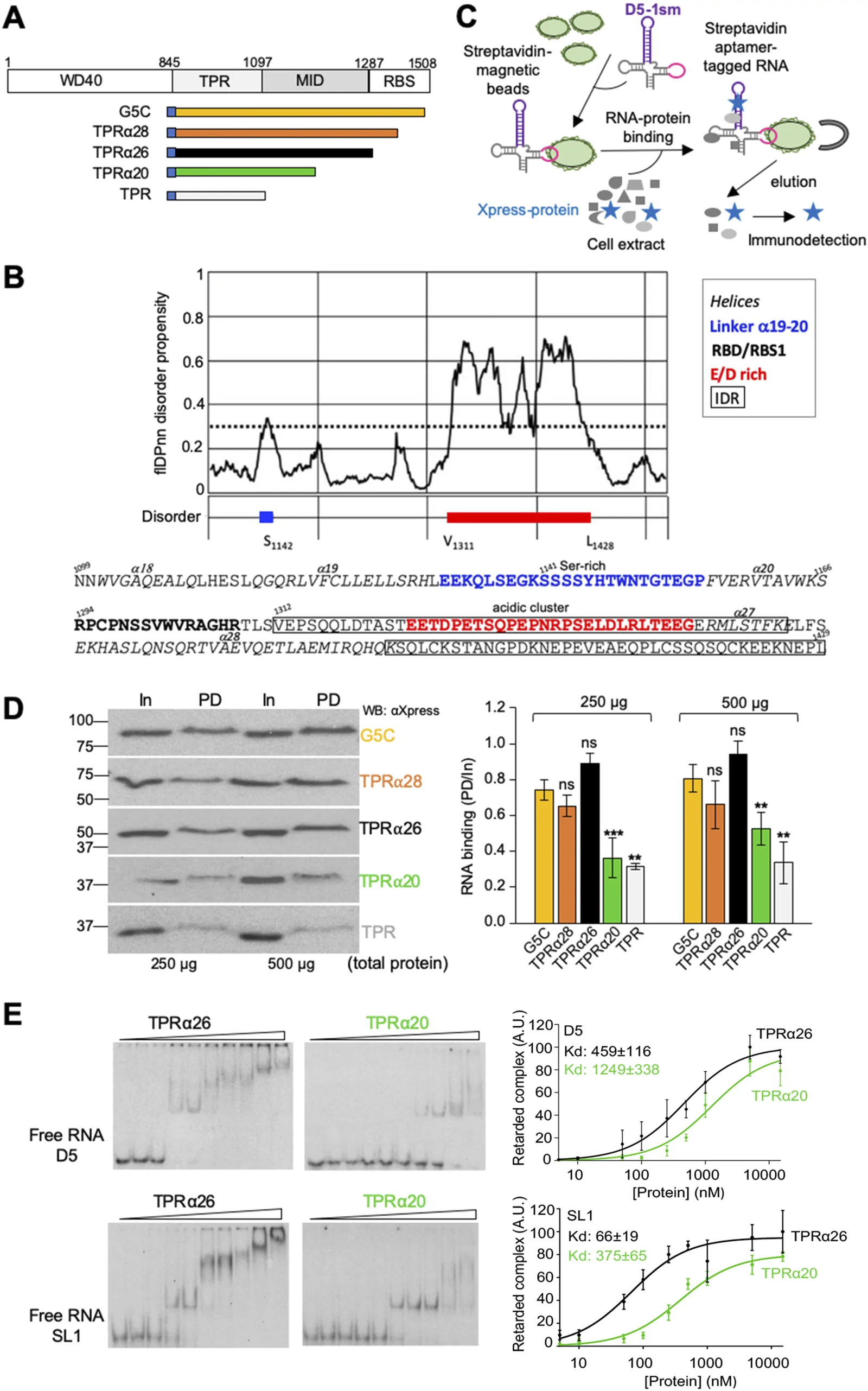
Identification of C-terminal sequences of GEMIN5 regulating RNA binding. **(A)** Diagram of the GEMIN5 domains. Numbers mark the boundaries of the WD40 domain, the TPR dimerization module, the helical regions 18–20 and 21–26, and the RBS domain. The Xpress-tagged proteins expressed from G5C (845–1508), TPRα28 (845–1436), TPRα26 (845–1286), TPRα20 (845–1170), and TPR (845–1097) are aligned below the diagram. **(B)** GEMIN5 disorder propensity, defined with flDPnn. The positions of the flexible loop and the acidic cluster are highlighted by blue and red bars, respectively. The sequence below the graph indicates the sequence and position of residues encompassing the serine-rich motif and the acidic cluster, flanked by the indicated helices. Bold black letters denote RBS residues known to mediate RNA binding. **(C)** Schematic representation of the streptavidin aptamer-tagged RNA (D5-1sm) and overview of the Xpress-protein capture procedure. **(D)** RNA binding of Xpress-tagged GEMIN5 fragments (G5C, TPRα28, TPRα26, TPRα19-20, and TPR) to D5-1sm RNA using increasing amounts (250 and 500 μg) of soluble cytoplasmic extracts. Immunodetection of tagged GEMIN5 was verified using anti-Xpress antibody (right panel). Relative RNA binding intensity of GEMIN5 proteins bound to D5 RNA (pulldown/input) (left panel). Bars represent the protein intensity (mean ± SEM) of at least three independent experiments. Asterisks denote *P*-values (**P < 0.01, ***P < 0.001). **(E)** RNA binding in vitro. Representative examples of the gel-shift assays conducted with purified TPRα26 or TPRα20 proteins and uniformly labeled D5 or SL1 RNA. The graph represents the adjusted curves and Kds (nM) obtained from the quantification of three independent gel-shift assays with increasing amounts of TPRα26 or TPRα20 proteins. Source data are available for this figure.

According to the Cryo-EM structure, G5C (aa 845–1508) consists of three modules (24) (Fig 1A). At the N terminus, the TPR-like module, including 17 helices, folds as a homodimer (20). The middle (MID) region (aa 1097–1287) comprises helices 18–26, whereas the most C-terminal region (RBS) (aa 1288–1508) folds as a pentamer. Three regions were unstructured in the Cryo-EM model, a flexible loop between helices 19 and 20 (aa 1133–1155), and two additional regions, one between helices 26 and 27 (aa 1294–1349), and another between helices 28 and 29 (aa 1392–1429) (24). The three-dimensional Cryo-EM model of G5C shows an internal pocket, which includes residues of the TPR-like module and the RBS region belonging to different subunits of the protein dimer. Moreover, exposed basic amino acids mutants within the TPR-like moiety, near the RBS domain, reduced RNA binding, with no gross change of the G5C three-dimensional structure (24). Since the TPR-like module alone does not bind RNA (20), it was proposed that two adjacent protomers are coordinated after pentamerization to bind RNA. Nevertheless, the contribution of each separate G5C modules for RNA binding remains to be elucidated.

GEMIN5 oligomerization allows the protein to assemble different complexes in the cellular context, depending on the version of the expressed protein (25). Along with its capability to interact with RNAs and ribosomes, this feature facilitates the assembly of diverse ribonucleoprotein (RNP) complexes, mostly involved in spliceosome assembly and translation control (25, 26). Mutants affecting distinct regions of the protein preferentially compromise the assembly of distinct RNP complexes, indicating the presence of dedicated domains involved in the recognition of specific partners, RNA, or proteins.

Further supporting the essential role of GEMIN5 in key RNA binding processes, it has been recently shown that biallelic variants in the *Gemin5* gene lead to neurodevelopmental disorders (27, 28, 29). These patients show phenotypic features apart from those originating from the SMN protein defects, causing spinal muscular atrophy (SMA) (30, 31, 32). The difference in the phenotypic disorders among SMA and GEMIN5 patients suggests the particular involvement of GEMIN5 in RNA processes beyond those altered by low levels of SMN protein. Importantly, nearly all the pathological variants affect the TPR-like moiety (33, 34, 35, 36, 37), highlighting the critical role of GEMIN5 oligomerization to perform its functions. Nonetheless, variants within the C-terminal region also lead to GEMIN5 malfunction, pointing towards the functional relevance of the RBS region.

Here we sought to analyze the presence of distinct RBSs within the C-terminal part of this multifunctional protein. We show that the RBD of this protein encompasses an extended binding surface, including conserved residues present in separate regions of the protein, a serine-rich motif within a flexible loop near the TPR-like moiety and an acidic cluster within the RBS domain. Furthermore, we show that specific modules within the C-terminal helical region of GEMIN5 modulate RNA binding, recruitment of the endogenous protein, ribosome binding, and translation regulation, underscoring a molecular basis for RNA regulation.

## Results

### Separate helical regions of GEMIN5 contribute RNA binding in total cell extracts

GEMIN5 comprises two different RBDs. The WD40 domain recognizes the U-rich sequence of snRNAs (18), whereas the RBS region binds mRNAs in a structure-dependent manner (38) (Fig 1A). Our previous work identified four positively charged amino acids within the TPR-like moiety whose mutants diminished the RNA binding ability of G5C (24). However, the likelihood of additional RBSs within this region of the protein was not explored. Given that the TPR-like module of GEMIN5 is a potent dimerization domain (20), it is plausible to propose that although the TPR-like alone does not interact with RNA in vitro, it may facilitate the proper folding to the remaining regions of the protein, presumably exposing hidden RBSs.

G5C encompasses several helical domains, including the TPR-like (helices 1–17), followed by a MID region, which comprises helices 18–26 (24). Another tract of helices, from 27 to 32, forms part of the RBS domain (22). Computational analysis of disorder propensity defined by flDPnn (39, 40) predicted the presence of a flexible loop between helices 19 and 20, characterized by a stretch of four consecutive serine amino acids, and a heavily disordered region between helices 26 and 27. In addition, the IDR region contains a cluster of acidic residues (Fig 1B), immediately downstream of aromatic and basic residues which determine the RNA binding ability of a short version of the protein, termed RBS1 (23). The potential involvement of each of these flexible regions in regulating RNA binding was not known. Therefore, we set up to explore the possibility that an extended TPR region could also be involved in RNA binding.

To systematically analyze the RNA binding capability of distinct G5C regions, we expressed Xpress-tagged proteins covering consecutive helical motifs in HEK293 cells (Fig 1A). Then, we used streptavidin magnetic beads coated with a streptavidin aptamer-tagged D5 RNA, a structural domain of the IRES element previously shown to interact with GEMIN5 (41, 42). The streptavidin aptamer fused to the RNA element of interest allows streptavidin high-affinity pulldown (43, 44). Consequently, this aptamer facilitates measurement of proteins bound to the aptamer-tagged RNA. After elution of the bound proteins and Xpress immunodetection, this procedure enabled the RNA binding quantification of Xpress-tagged proteins (Fig 1C). A comparative study of serially truncated proteins, including G5C, TPRα28, TPRα26, TPRα20, and TPR, revealed the interaction of G5C with D5-tagged RNA in the competitive environment of total cell extracts (Fig 1D), confirming previous in vitro binding data (24). In comparison to G5C, the proteins TPRα26 and TPRα28 retained the capacity to recognize RNA, whereas TPRα20 showed a reduced but dose-dependent RNA binding ability. In contrast, the basal RNA binding intensity of the TPR moiety was dose independent (Fig 1D). These data suggest that the flexible loop between helices 19 and 20 is crucial to facilitate RNA binding in the competitive cellular environment.

The RNA binding activity of TPRα20 was unforeseen because the purified TPR protein was unable to bind in vitro several RNA probes differing in sequence and structure (20). Therefore, to further verify the RNA binding ability of TPRα26 and TPRα20, we purified the proteins to measure RNA binding in vitro using two probes, D5 and SL1 (Fig 1E), previously characterized as GEMIN5 targets (22). Confirming the data observed using total cell extracts, both proteins showed a dose-dependent RNA binding ability, although TPRα20 displayed lower affinity than TPRα26 with both probes (Fig 1E). These data confirmed that residues within the region 1097–1170 (helices 18–20) confer RNA binding to GEMIN5 cognate targets. In addition, the higher RNA binding ability exhibited by TPRα26 indicated that residues spanning the α21–α26 region contribute as well.

**Figure S1.**
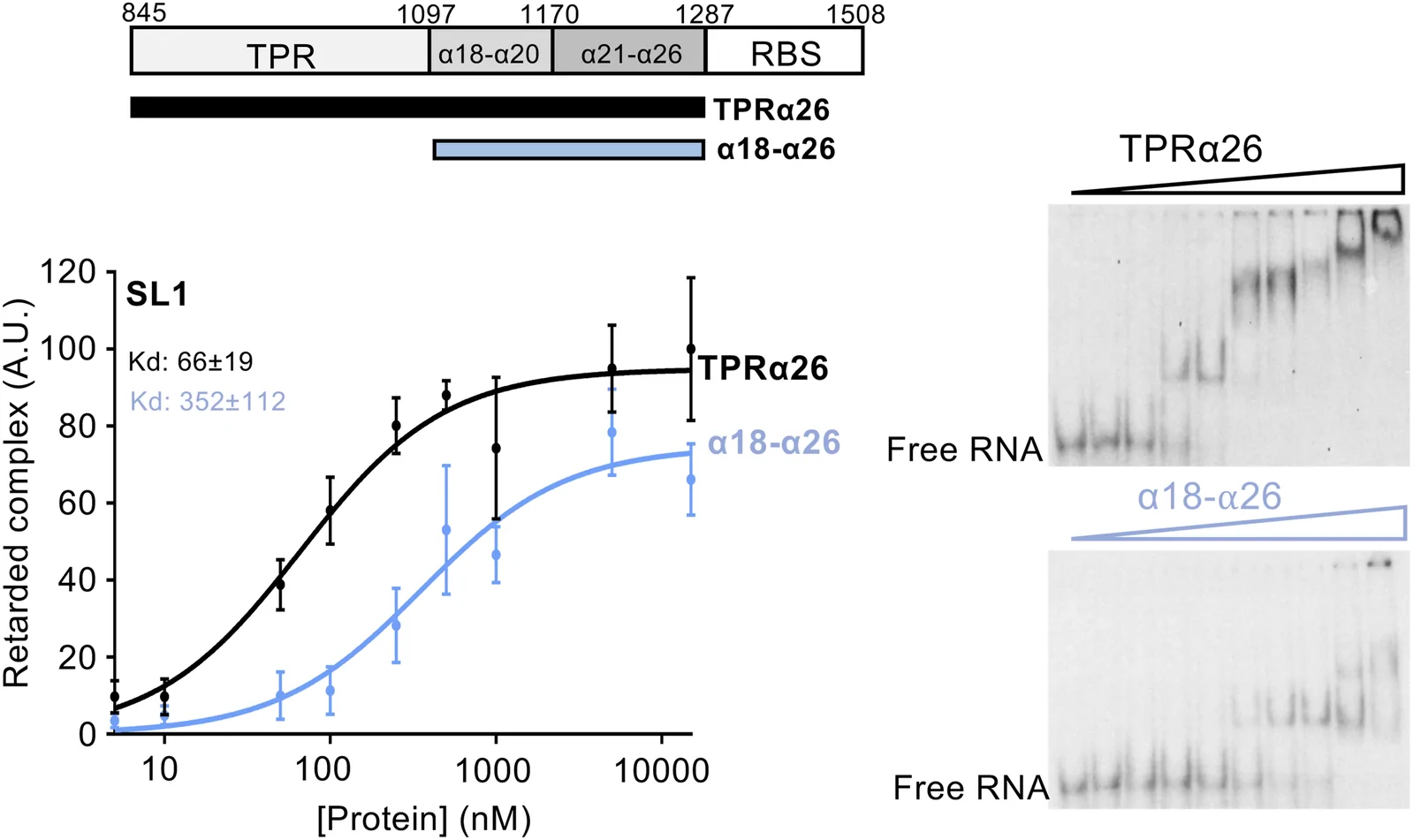
RNA binding in vitro. Representative examples of the gel-shift assays conducted with purified TPRα26 or α18-α26 proteins and uniformly labeled SL1 RNA. Gel-shit image used for TPRα26 is the same image used in Fig 1E. Graph representing the adjusted curves and Kds (nM) obtained from the quantification of three independent gel-shift assays.

To reinforce the contribution in RNA binding of the G5C helical regions independent of the oligomerization mediated by the TPR-like module, we generated the protein α18–26 to perform RNA binding assays in vitro. In comparison to its counterpart TPRα26, the protein α18–26, lacking the TPR-like moiety, retained a mild RNA binding capacity, differing in the number and mobility of the retarded complexes (Fig S1), further supporting that a separate helical region within G5C is involved in RNA binding.

### The acidic cluster of the IDR is implicated in RNA recognition

**Figure 2.**
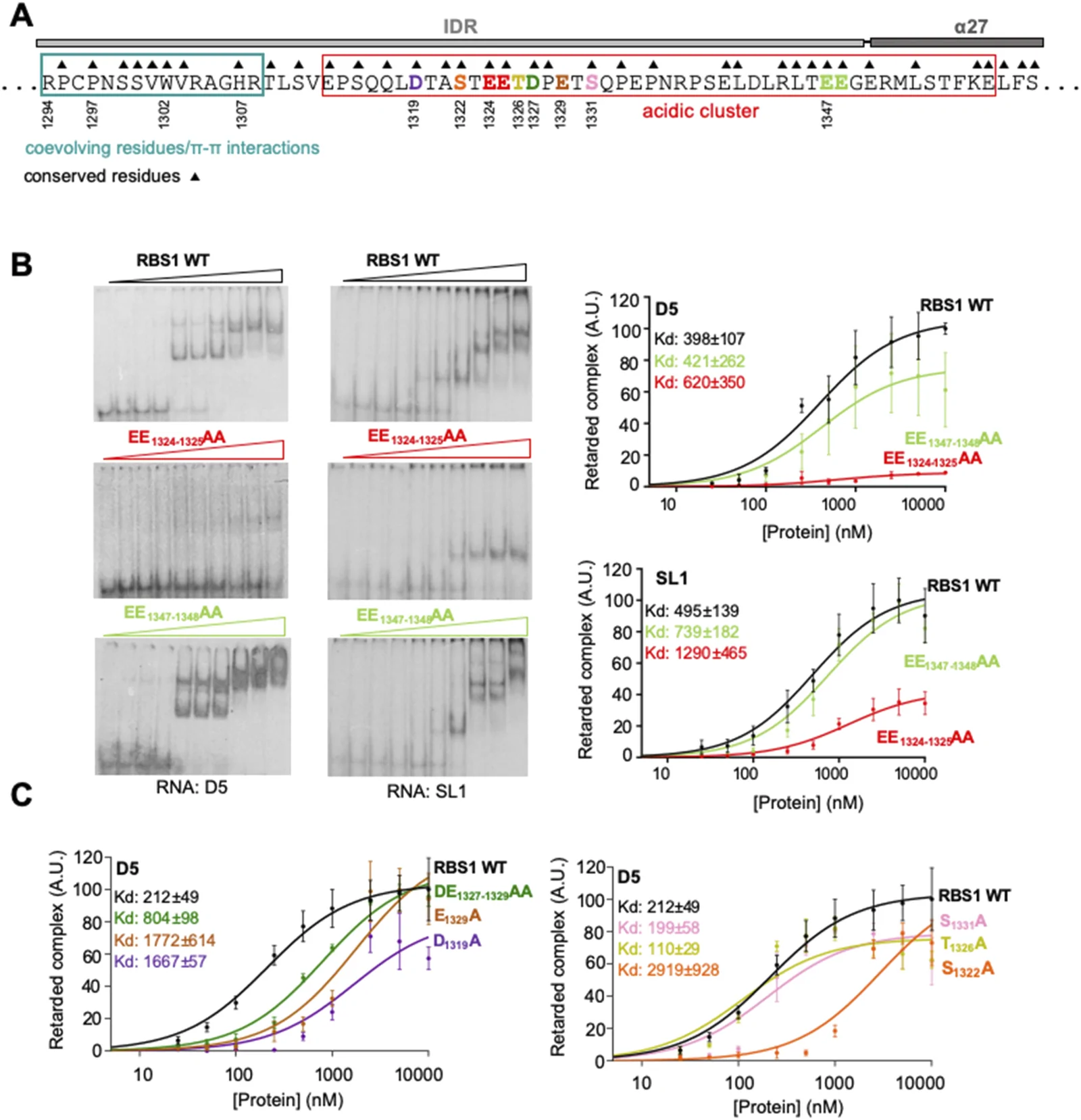
RNA binding studies of the acidic cluster. **(A)** Amino acid sequence of the IDR. Blue or red boxes denote residues involved in π–π interactions with RNA or the acidic cluster, respectively. Black triangles indicate conserved residues. Numbers mark positions substituted with alanine. **(B)** RNA binding in vitro of proteins mutants with amino acid substitutions in selected positions of the acidic cluster. Representative examples of gel-shift assays conducted with RBS1 wild type (WT), or mutants EE_1324–1325_AA, EE_1347–1348_AA, and D5 or SL1 probes. Graph representing the adjusted curves and Kds (nM) obtained from the quantification of three independent gel-shift assays labeled RNAs incubated with increasing amounts of RBS1 WT or the indicated mutants. **(C)** Adjusted curves and Kds (nM) obtained from the quantification of three independent gel-shift assays labeled RNAs incubated with increasing amounts of RBS1 WT or mutants carrying substitutions in additional conserved aspartic or glutamic residues (D_1319_A, D_1327_A/E_1329_A, and E_1329_A) (left panel), and serine and threonine residues (S_1322_A, T_1326_A, and S_1331_A) (right panel). Source data are available for this figure.

Earlier work reported the RNA binding capability of G5C (24), and RBS1 fragment (aa 1287–1412) (22) to D5 and SL1 probes. The amino acids tract RPCPNSSVWVRAGHR (Fig 2A), located at the N terminus of RBS1, constitutes a minimal motif that mediates RNA binding by establishing π–π interactions (23). This motif is located upstream of a conserved acidic cluster within the IDR (Fig 2A), whose contribution to RNA recognition remains unknown.

**Figure S2.**
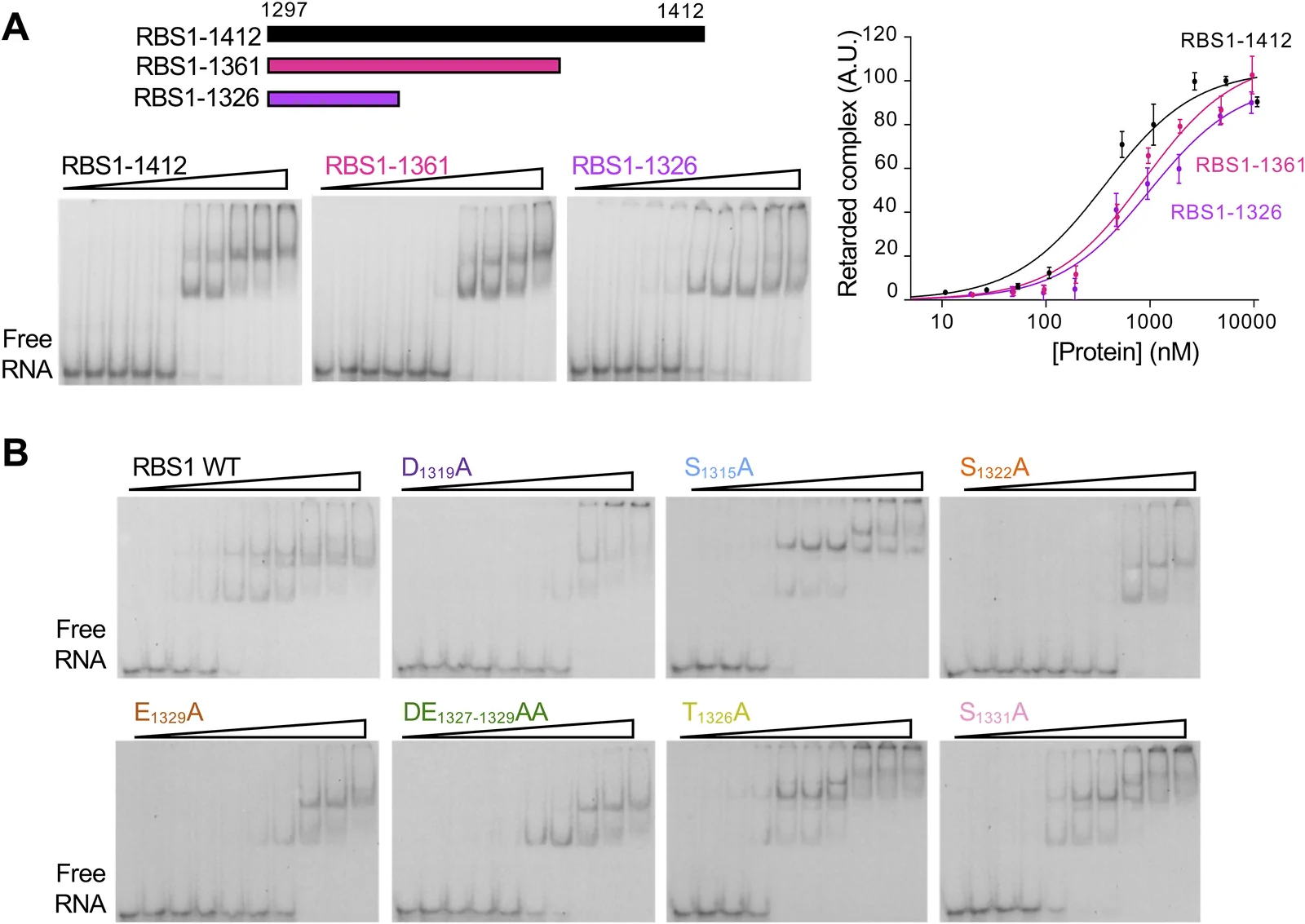
RNA binding in vitro. **(A)** Gel-shift assays conducted with purified RBS1 protein and the indicated fragments with uniformly labeled RNA. Graph representing the adjusted curves obtained from the quantification of three independent gel-shift assays. **(B)** Representative examples of the gel-shift assays conducted with purified RBS1 WT protein and the indicated mutants and uniformly labeled SL1 RNA.

Given that part of the IDR forms an internal pocket in the G5C structure facing basic amino acids within the TPR-like moiety (24), we sought to assess the influence of conserved acidic residues of the IDR for RNA recognition using D5 and SL1 probes, previously reported to be recognized by the RBS region (23). To this end, we generated substitutions in glutamate pairs 1324–1325 and 1347–1348, to alanine. The RNA binding intensity of the mutant proteins was significantly diminished in the first case EE_1324–1325_AA, but not in the second one EE_1347–1348_AA, with both probes (Fig 2B) suggesting that EE_1324–1325_ is involved in RNA recognition. Further supporting this conclusion, the RNA binding activity of the truncated fragments RBS_1–1326_ and RBS_1–1361_ (Fig S2A) indicated that a short protein, harboring residues 1294–1326 is sufficient to promote RNA binding in vitro.

To test the contribution of residues next to EE_1324–1325_ to RNA binding, we generated variant proteins carrying substitutions of conserved acidic and polar amino acids (D, E, S, and T, located between positions 1319–1331) (Fig 2A) to alanine. The results derived from the protein mutants indicated that, individually, S_1315_, S_1322_, and D_1319_ decreased the RNA binding capacity of the protein (Figs 2C and S2B), suggesting that residues immediately downstream of the minimal RBS motif regulate RNA binding.

### Conserved serine residues within the flexible loop downstream of the TPR-like impact RNA binding

**Figure 3.**
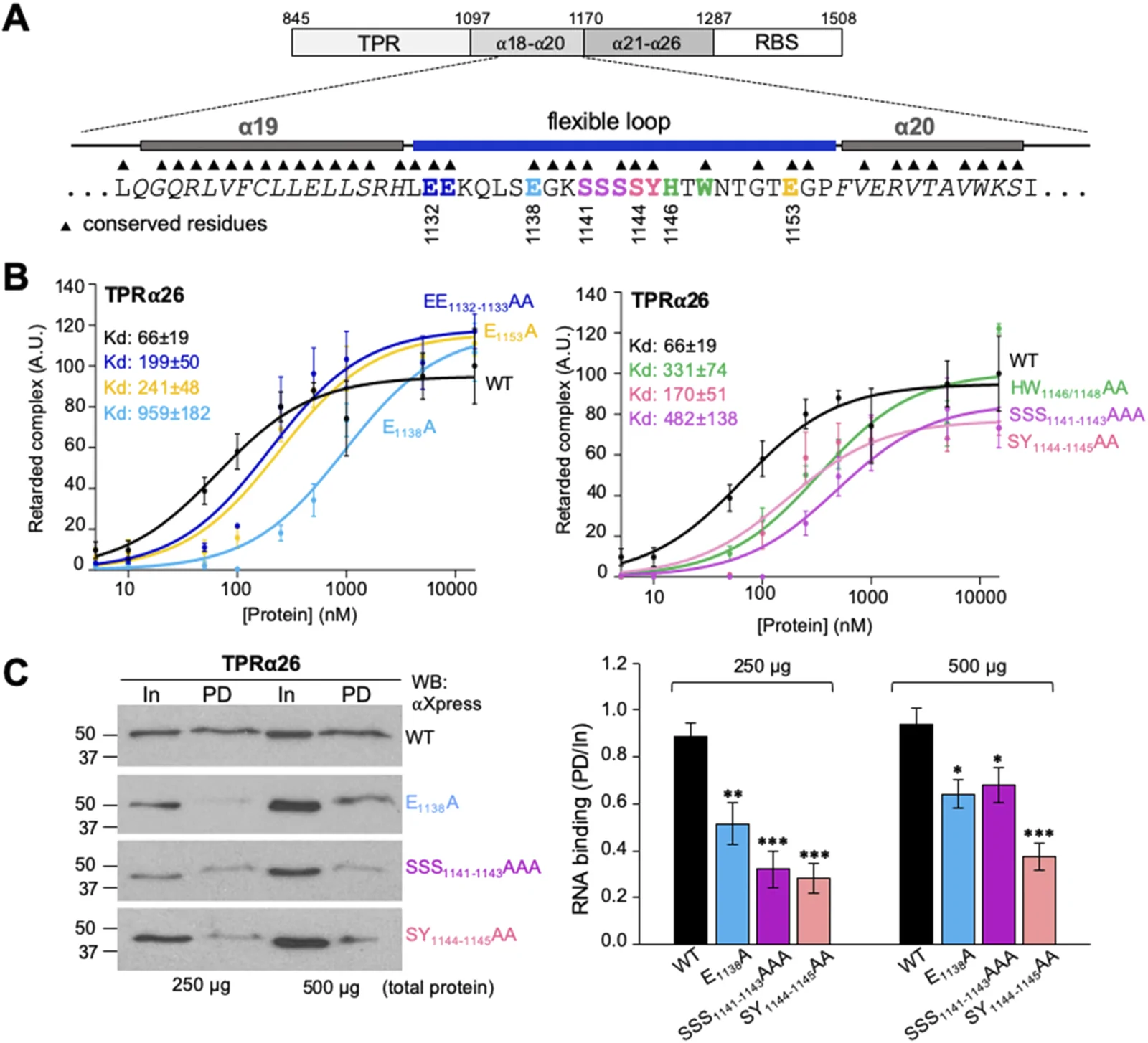
RNA binding studies of the flexible loop located between helices 19–20. **(A)** Amino acid sequence of the flexible loop. Black triangles indicate conserved residues. Numbers denote positions substituted with alanine. **(B)** Graphs depicting the relative RNA binding ability in vitro and Kds (nM) of proteins carrying substitutions in conserved residues (serine, glutamic, histidine, or tryptophan) to alanine compared with the WT protein TPRα26. **(C)** RNA binding of Xpress-tagged TPRα26 and the mutants E_1138_A, SSS_1141–1143_AAA, or SY_1144–1145_AA to D5-1sm RNA using increasing amounts of total cytoplasmic extracts. Immunodetection of tagged proteins was verified using anti-Xpress antibody (left panel). Relative RNA binding intensity of GEMIN5 bound to D5 RNA (pull-down/input) (right panel). Bars represent the protein intensity (mean ± SEM) of three independent experiments. Asterisks denote *P*-values (*P < 0.05, **P < 0.01, ***P < 0.001). Source data are available for this figure.

**Figure S3.**
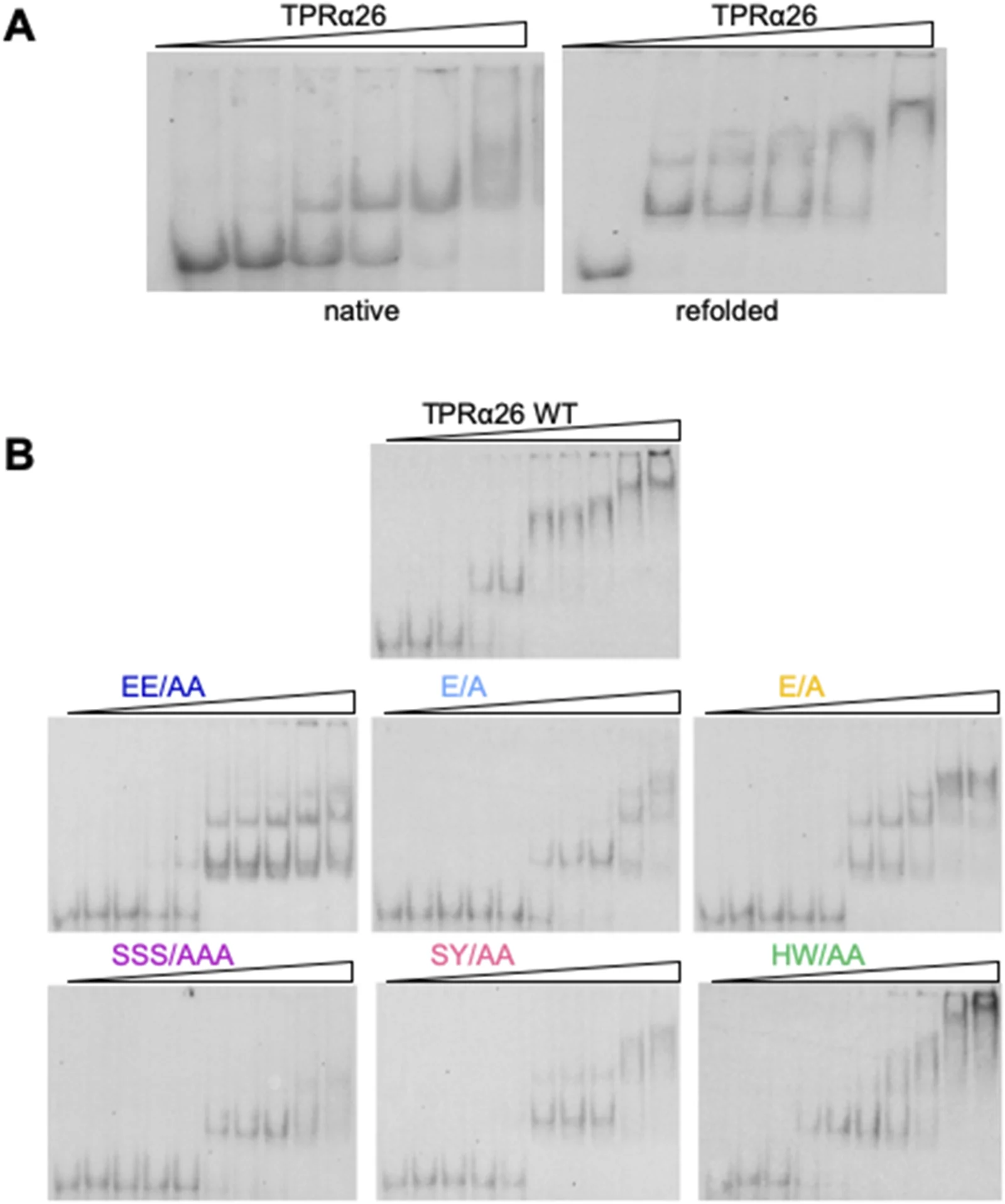
RNA binding in vitro. **(A)** Gel-shift assays conducted with TPRα26 WT protein purified under native (left) or denaturing conditions followed by refolding in elution buffer supplemented with 1 mM DTT (right). **(B)**. Representative examples of the gel-shift assays conducted with purified TPRα26 WT and the indicated mutant proteins and uniformly labeled SL1 RNA.

Taking into consideration the previously reported G5C structure, RNA binding can be promoted by an internal pocket facing basic residues of the TPR-like module with two flexible regions (24) located within helices α19–20 and α26–27 (Fig 1B). The first one comprises conserved glutamate, serine, tyrosine, and tryptophan residues (Fig 3A), present in TPRα20 protein, which displayed RNA binding in vitro and in cell extracts (Fig 1D and E). Therefore, we generated mutants within the linker between helices α19–20, substituting these conserved residues, individually or combined, to alanine (Fig 3A). First, as a control of RNA binding, in vitro binding assays performed using TPRα26 protein, purified under native and denatured conditions followed by refolding in elution buffer supplemented with 1 mM DTT, resulted in similar RNA binding ability (Fig S3A). Interestingly, the single mutant E_1138_A, the triple substitution of the Ser-rich motif SSS_1141–1143_AAA, as well as the double mutant SY_1144–1145_AA reduced RNA binding. In contrast, the double substitutions EE_1132–1133_ to Ala and HW_1146/1148_ to Ala did not affect RNA binding in vitro (Figs 3B and S3B). Furthermore, the RNA binding assays performed in the competitive context of a total cell extract expressing the mutant proteins E_1138_A, SSS_1141–1143_AAA, and SY_1144–1145_AA revealed statistically significant differences with the WT protein (Figs 3C, and S3B). Therefore, we conclude that the amino acid tract EGKSSSSYH, located at the center of the flexible loop between helices 19–20, regulates RNA binding to cognate RNAs.

**Figure 4.**
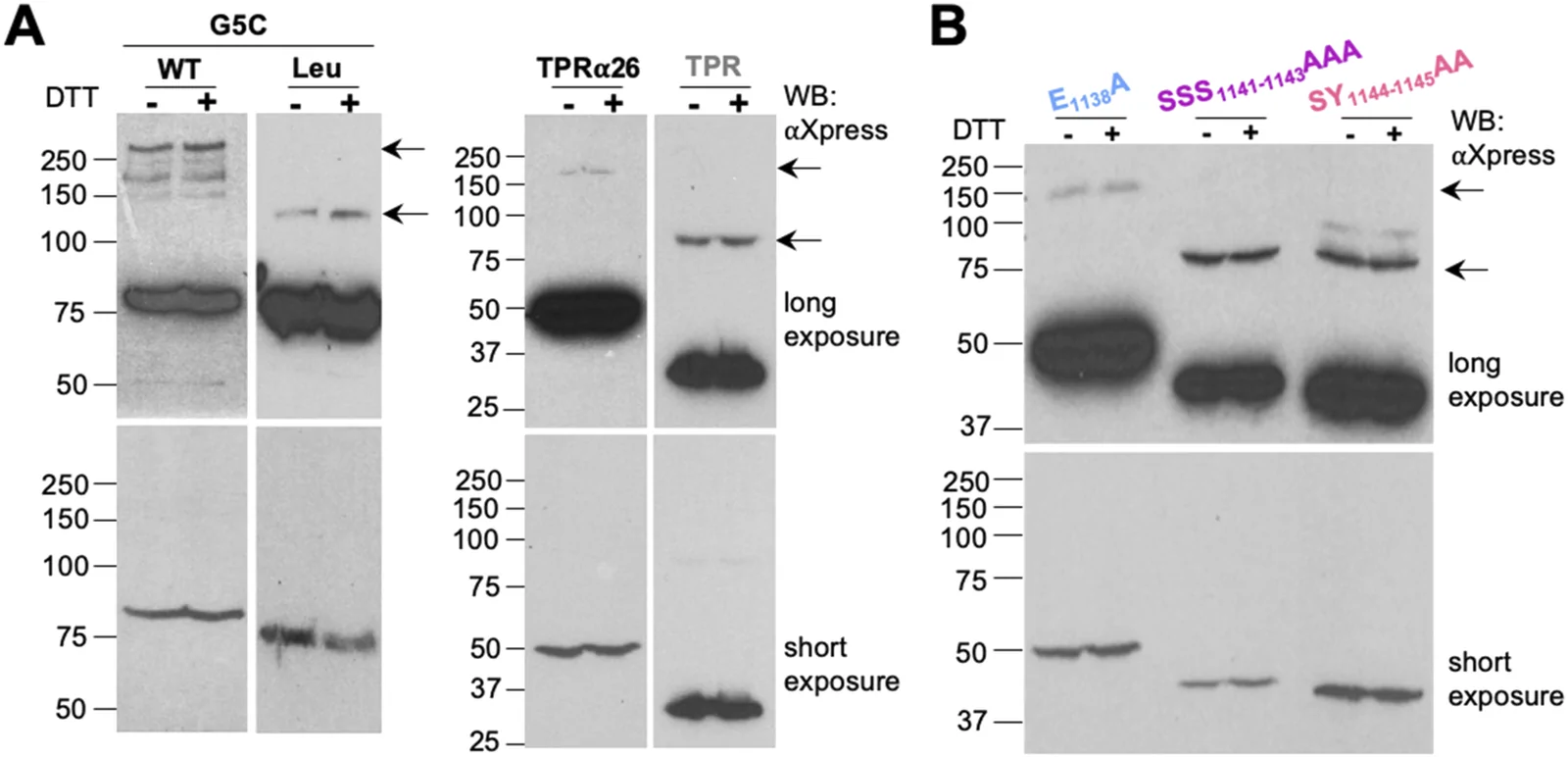
Assembly of SDS-resistant oligomers by GEMIN5 protein mutants. **(A)** SDS-resistant oligomer assembly by GEMIN5 fragments. HEK293 cells expressing the Xpress-His constructs (G5C, G5C-Leu, TPRα26, or the TPR alone) in independent triplicate assays were lysed and stored at −70°C until used. For SDS–PAGE, the samples were treated with SDS-loading buffer supplemented, or not, with extra amounts of DTT (5 mM) and heated 3 min at 92°C. Western blot analysis was carried out using anti-Xpress antibody. Light exposure is shown to visualize the Xpress-His constructs, long exposure detects high molecular weight bands. **(B)** SDS-resistant oligomer assembly of TPRα26 mutants. Extracts prepared from HEK293 cells expressing the Xpress-His constructs (E_1138_A, SSS_1141–1143_AAA, and SY_1144–1145_AA) were analyzed side by side; the control blot of the WT TPRα26 is shown in (A). Source data are available for this figure.

Because the above analyzed proteins contain the TPR-like moiety, it was plausible that they could oligomerize in solution, presumably affecting RNA binding properties. To test this possibility, we analyzed the presence of SDS-resistant assemblies in cellular lysates expressing these proteins, both in the presence and absence of extra DTT (5 mM). These conditions have been previously established for the oligomerizing proteins TRIM75, RIG-I, or Tau, among others (45, 46, 47). First, as a control of the assembly of SDS-resistant oligomers, we used the extended C-terminal region G5C and the short TPR-like moiety, previously shown by Cryo-EM and X-ray structure to form a dimer of pentamers and a dimer, respectively (20, 24, 25). Beyond the monomeric version of the protein observed in short exposure times, long exposure of the same immunoblot allowed the detection of retarded SDS-resistant assemblies in the case of G5C (Fig 4A), suggesting the formation of stable oligomers. In support of this conclusion, a parallel analysis of the leucine core mutant G5C-Leu (L_1381_D, M_1384_D, I_1385_D) which abolished the assembly of the pentameric structure (24), disclosed the formation of a complex with the apparent mobility of a dimer in long exposures, rather than higher Mw oligomers. Similar analysis of cell extracts expressing TPRα26 or TPR proteins, which do not have the domain adopting a pentameric structure, showed a weak but detectable oligomer with apparent mobility close to a dimer (Fig 4A).

Next, we verified the ability of the mutant TPRα26 proteins to form SDS-resistant oligomers compared with the wild type version. Immunodetection of these proteins revealed the presence of the corresponding monomeric protein (short exposure). The mutants SSS_1141–1143_AAA and SY_1144–1145_AA displayed a faster mobility in SDS–PAGE compared to WT and E_1138_A proteins, likely due to changes in their amino acid composition. Interestingly, a longer exposure of the same immunoblot revealed the presence of higher Mw complexes, compatible with the assembly of dimeric complexes in all proteins. In comparison with the WT TPRα26 protein, the mutants SSS_1141–1143_AAA and SY_1144–1145_AA produced more intense apparent dimers (Fig 4B), suggesting that mutants on the Ser-rich motif within the flexible loop may favor self-dimerization.

**Figure 5.**
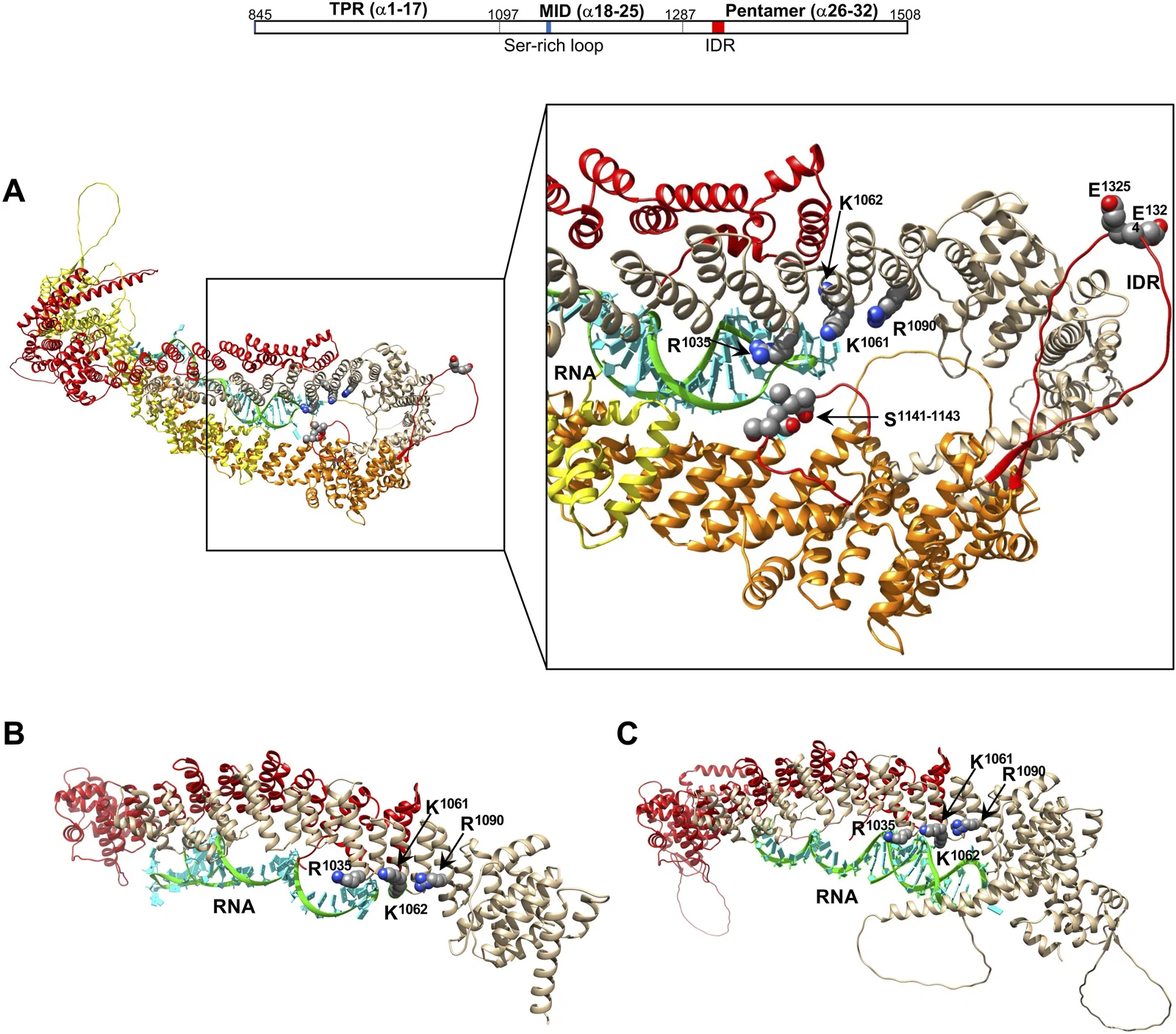
Structural model of G5C associated with D5 RNA with AlphaFold. **(A)** Predicted structure of G5C tetramer RNA. Red, yellow, grey, or orange denote the subunits. D5 RNA is shown in blue. **(B)** Predicted structure of TPRα26–RNA dimer. **(C)** Structure of G5C–RNA dimer. Structural elements in the AlphaFold image were highlighted using UCSF Chimera X (48).

Our previous structural data revealed the impact of protein oligomerization for RNA binding (24). Therefore, to reconcile the SDS-resistant oligomer data with the CryoEM structure of G5C, we generated a model of the G5C protein in the presence of D5 RNA. Interestingly, AlphaFold modeling of G5C–RNA shows D5 RNA surrounded by residues S_1141–1144_ of one subunit and R_1035_ and K_1061_ within the TPR-like module of the other subunit (Fig 5A). Furthermore, modeling of the TPRα26 protein suggests the assembly of an elongated dimer engulfing the RNA via residues R_1090_, K_1061_, and R_1035_ of the TPR-like moiety (Fig 5B), further supporting the involvement of the ser-rich motif for RNA binding.

### RNA binding and self-dimerization negatively impact on GEMIN5 recruitment and ribosome binding

**Figure 6.**
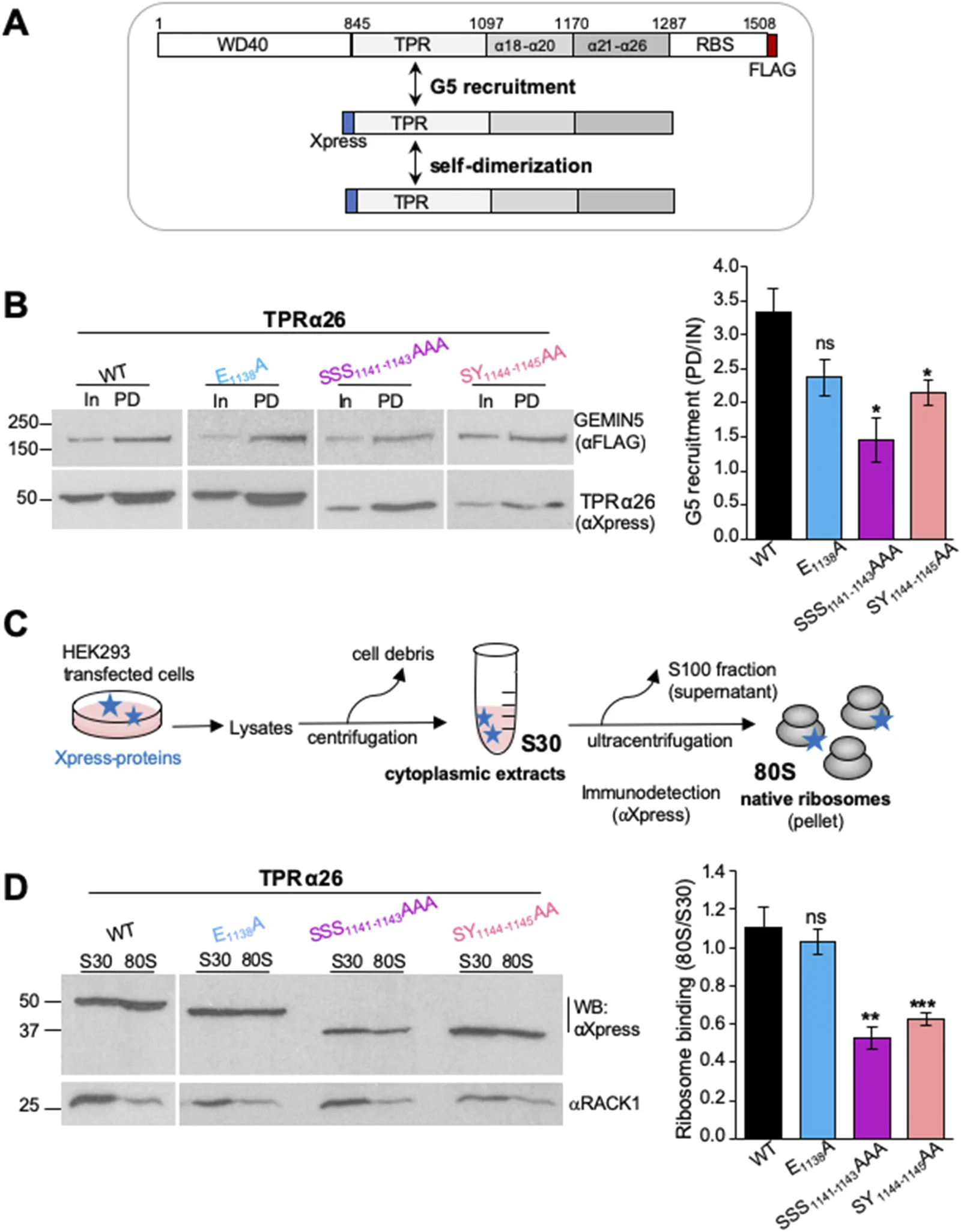
GEMIN5 oligomerization determines ribosome binding and translation regulation. **(A)** Diagram representing the involvement of the TPR module in recruiting the FLAG-tagged GEMIN5 protein from total cytoplasmic extracts (G5 recruitment), and the Xpress-tagged TPRα26 (self-dimerization). **(B)** Impact of the flexible loop in G5 recruitment. HEK293 cells expressing G5-FLAG and the indicated Xpress-His TPRα26 constructs were lysed and subjected to pull-down assays. Protein expression (In, inputs) and pull-down (PD) samples of independent experiments were analyzed by Western blot (WB) using anti-FLAG and anti-Xpress antibodies (left panel). Histograms depicting G5 recruitment measured by relative intensity (PD/In) of G5-FLAG associated with the Xpress-His TPRα26 WT and mutant proteins (right panel). The intensity of the bands was normalized by the PD/In ratio obtained in control cells performed side by side. Values represent the mean ± SEM, asterisks denote *P*-values (ns, not significant, *P < 0.05). **(C)** Association of TPRα26 with native ribosomes. Overview of the cell fractionation procedure. **(D)** HEK293 cells transfected with Xpress-His TPRα26 wild type or the indicated mutants were processed to isolate native ribosomes (80S). The protein levels present in S30 fractions (cytoplasmic lysates) and 80S ribosomes were determined using anti-Xpress antibody. Anti-RACK1 was used as loading control (left panel). Graph representing relative ribosome binding of Xpress-TPRα26 WT or the indicated mutants, in native ribosomes (80S) divided by the intensity present in total lysates (S30). Values represent the mean ± SEM, asterisks denote *P*-values (**P < 0.01, ***P < 0.001). Source data are available for this figure.

Our previous data have shown a strong impact of protein oligomerization on the ability of expressed GEMIN5 to recruit the endogenous protein (25). However, the capability to form oligomers could also be affected by the ability to interact with RNA. To test this hypothesis, we proceeded to analyze the influence of TPRα26 mutants on the competence to form oligomers with the endogenous protein and therefore, to recruit GEMIN5 (Fig 6A).

The full-length GEMIN5 protein, fused to a FLAG tag at the C terminus, and the Xpress-TPRα26 wild-type and mutant proteins were individually expressed in HEK293 cells. Expression of all proteins was verified by Western blot in Input samples (In) (Fig 6B). The ratio of pull-down/Input of the FLAG-tagged GEMIN5 protein was analyzed to verify the capacity of the Xpress-His proteins to recruit GEMIN5. The association with GEMIN5-FLAG was observed in the wild type TPRα26, consistent with the presence of an active TPR-like moiety. Relative to the WT TPRα26, the mutants SSS_1141–1143_AAA and SY_1144–1145_AA, revealed a diminished ability to recruit GEMIN5-FLAG (Fig 6B), whereas E_1138_A showed a nonsignificant decrease. These data are inversely correlated with the self-dimerization behavior of these mutants. Thus, we suggest that the mutant proteins SSS_1141–1143_AAA and SY_1144–1145_AA were compromised in both activities, RNA binding and oligomerization dynamics.

Next, because GEMIN5 oligomerization failure has been correlated with diminished ribosome binding (25), we tested the ribosome binding intensity of the TPRα26 mutants (Fig 6C). Relative to the WT protein, both SSS_1141–1143_AAA and SY_1144–1145_AA showed a decreased 80S/S30 intensity, whereas the difference between WT and E_1138_A was not significant (Fig 6D). Therefore, these mutant proteins behaved similarly in G5 recruitment, self-dimerization, and ribosome binding.

**Figure S4.**
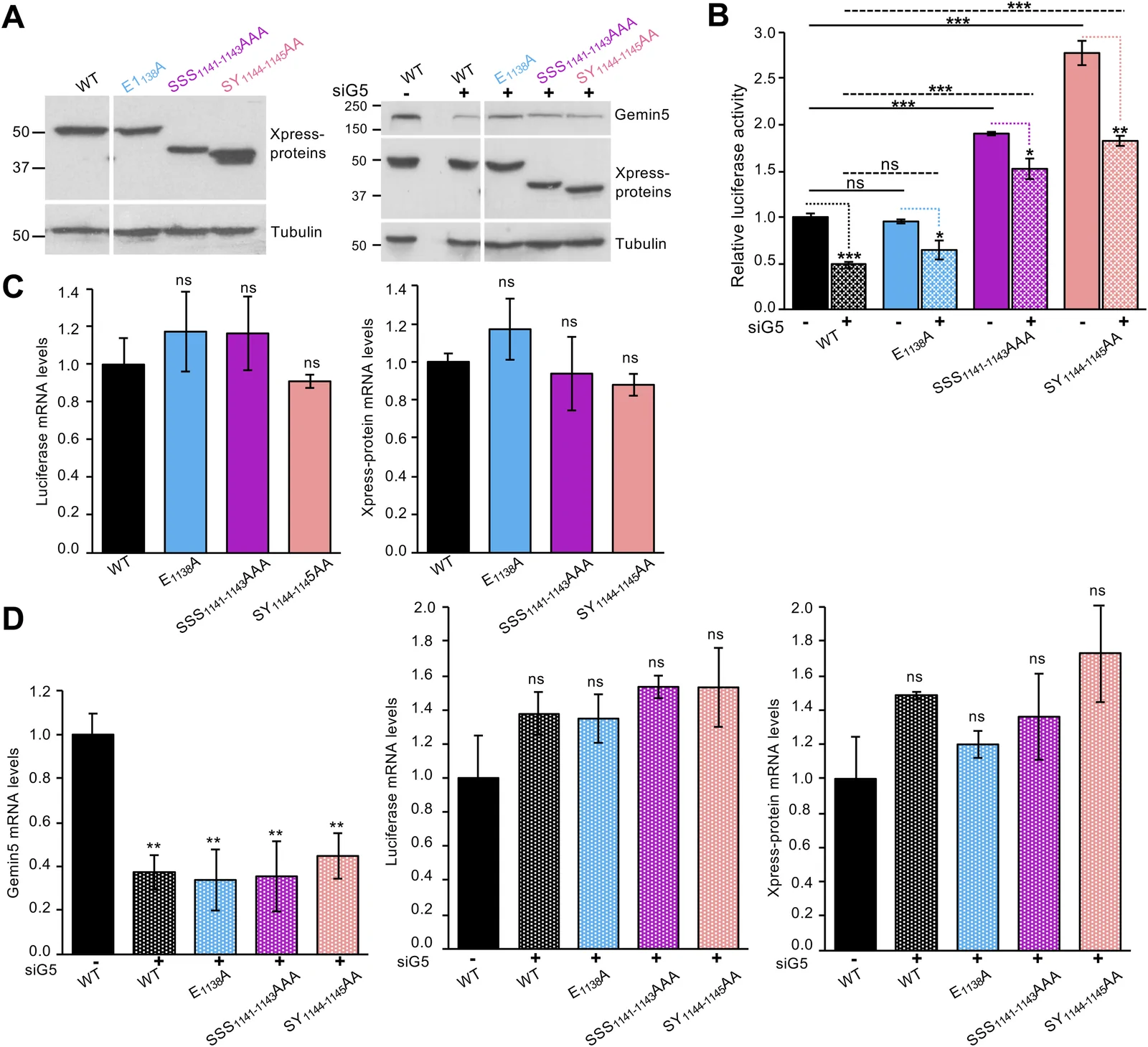
Translation regulatory effects by GEMIN5 mutants. **(A)** Cap-dependent translation activity determined in control HEK293 cells, or siG5 silenced cells, expressing Xpress-TPRα26 WT, the mutant E_1138_A, SSS_1141–1143_AAA, or SY_1144–1145_AA co-transfected with pCAP-luc plasmid. Protein expression was monitored by WB using anti-Xpress. Tubulin was used as the loading control. **(B)** Relative luciferase activity observed in control cells (−) and siG5-RNA silenced cells (+) expressing TPRα26 WT or the indicated mutants. Values are normalized to cells expressing Xpress-TPRα26 WT conducted side by side. Values represent the mean ± SEM obtained in three independent assays. Asterisks denote *P*-values (*P < 0.05, **P < 0.01, ***P < 0.001). **(C)** Steady-state mRNA levels of luciferase reporter and Xpress-His proteins present in transfected cells at the time of harvesting determined by RT-qPCR. **(D)** Steady-state mRNA levels of Gemin5, luciferase mRNA reporter, and Xpress-His protein mRNAs present in G5 silenced transfected cells at the time of harvesting determined by RT-qPCR.

The different behavior of the proteins with substitutions on the Ser-rich motif led us to analyze the regulatory effect of these mutants in cap-dependent translation of RNA reporters encoding firefly luciferase, under normal and GEMIN5-silenced conditions (Fig S4A and B). Silencing of GEMIN5 induced a significant decrease in the stimulatory activity exerted by WT TPRα26 protein, suggesting that this effect could be related to the interactions between GEMIN5 and TPRα26. However, whereas the relative luciferase activity of the WT and E_1138_A was similar, the expression of SSS_1141–1143_AAA and SY_1144–1145_AA resulted in a significant increase in cap-dependent translation (Fig S4A and B). No significant changes in mRNA levels were observed under normal or GEMIN5-silenced conditions (Fig S4C and D). The fact that these mutants favored self-dimerization, but disfavored GEMIN5 recruitment and ribosome binding, suggests that the Ser-rich motif mutants can contribute to translation through a mechanism independent of the endogenous GEMIN5 protein.

## Discussion

A prominent feature of GEMIN5 is the oligomerization propensity, endowed by the TPR-like module located in the central region of the protein (20). Adding further complexity to this singular protein, the assembly of a pentamer structure involving residues of the C terminus of the protein, assisted by the TPR-like dimerization domain, generates a decamer architecture (24). How this dimer of pentamers is able to recognize distinct targets, either RNAs or proteins, is not fully understood. In this work we found that the RBD of GEMIN5 consists of an extended binding surface, comprising multiple helical motifs. Furthermore, RNA binding is regulated by two separate interhelical regions, a short flexible loop rich in serine residues near the dimerization module, and a distal unstructured region located within the RBS domain, enriched in negatively charged residues.

The oligomerization property observed in the Xpress-tagged half C-terminal G5C and the protein extended up to helix 26 appears to be associated with the capability to self-dimerize, but also with the ability to recruit the endogenous protein in the HEK293 cellular cytoplasm forming hetero-oligomers (25). This feature observed in non-neuronal cell extracts may have limitations due to the use of overexpressed constructs. However, this result appears to be independent of the tags added, as the oligomerization of the protein has been observed in different experimental conditions (24, 49). Further supporting the intrinsic oligomerization feature of GEMIN5, previous studies reported that the purified GEMIN5 elutes as high molecular weight polymers in gel filtration analysis, with the apparent mobility of a tetramer (49). Thereby, considering that the assembly of distinct oligomeric complexes, hence adopting distinct architectures, could expose separate RNA recognition motifs, we postulated that distinct versions of the G5C protein could exhibit different RNA binding potential. In agreement with this hypothesis, proteins encompassing the dimerization moiety followed by increased number of helical domains (TPRα20, TPRα26, and G5C), also showed enhanced RNA binding capacity in the context of soluble cellular extracts (Fig 1). Meanwhile, the TPR-like moiety alone, which was negative in RNA binding using in vitro assays (20), displayed basal RNA binding intensity under the same cellular extract conditions. These data led us to conclude that specific residues within the helices 19–20 facilitated RNA binding.

The RNA binding ability of TPRα26 was observed in the competitive context of total proteins, but also in vitro using purified components. In addition, TPRα26 forms SDS-resistant oligomers with lower intensity and faster mobility than G5C, showing the apparent mobility of a dimer. This observation led us to suggest that helices 18–26 promote the formation of an extended dimer in TPRα26.

### Dissecting RNA interaction mediated by an extended RNA-binding surface

In this study we have identified two different unstructured regions of GEMIN5 implicated in RNA binding, a serine-rich motif in a flexible loop between helices 19–20, and an acidic cluster within the IDR region of the RBS, between helices 26–27. Measurement of the individual impact of the serine-rich motif (EGKSSSSYH) and the acidic cluster (SQQLDTASTEETDPET) indicated that substitutions within each of these motifs to alanine confer a diminished RNA binding intensity in vitro. In support of the implication of each of these regions for RNA binding, the G5C three-dimensional structure revealed the presence of an internal pocket formed by two protein subunits, by which K and R positively charged residues within the helices 13, 15, and 17 of the TPR-like in one subunit are located in front of the flexible loop between helices 19–20 and the IDR on the other subunit (24).

**Figure S5.**
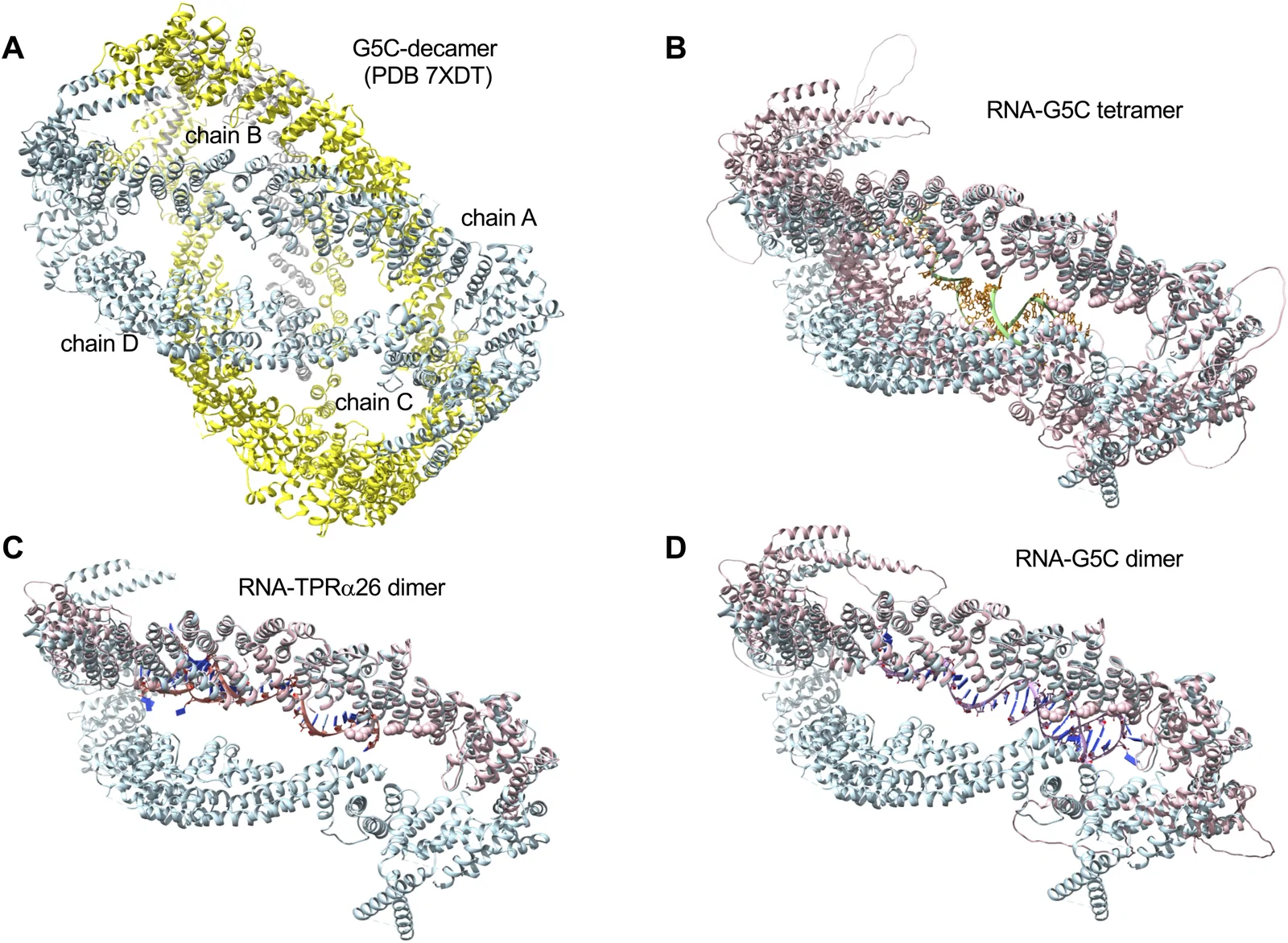
Structure of G5C dimer of pentamers (PDB 7XDT). **(A)** In blue, tetramer used to overlap the predicted structures of the AF models of the RNA–G5C tetramer complex (B); RNA–G5C dimer complex (C); RNA–G5C dimer (residues 845–1287) complex (D). Modeled structures are colored in pink.

The mechanism behind how these separate regions of GEMIN5 contribute to RNA binding remains elusive, although several hypotheses can be proposed. As mentioned above, the three-dimensional structure of the GEMIN5 C-terminal region adopts a homodecamer architecture comprising of a dimer of pentamers (PDB 7XDT). However, in those structures, the loops between helices 19–20 and 26–27 containing the serine-rich motif and the acidic cluster, respectively, were structurally unresolved because of their intrinsic flexibility. Interestingly, AlphaFold modeling (50, 51) of the G5C protein in the presence of D5 RNA showed a G5C tetramer structure that overlaps with chains A, B, C, and D of the Cryo-EM structure (24), whereas the G5C and TPRα26 dimers fit perfectly with chains A and B (Fig S5). More importantly, the G5C–RNA AlphaFold model shows a hypothetic structure in which D5 RNA is surrounded by residues R_1035_ and K_1061_ within the TPR-like module of one subunit and the S_1141–1144_ motif of the other subunit (Fig 5A).

Further supporting the involvement of the ser-rich motif for RNA binding, AlphaFold structural predictive modeling of the TPRα26 protein suggests the assembly of an elongated dimer engulfing the RNA via residues R_1090_, K_1061_, and R_1035_ of the TPR-like moiety (Fig 5B). The RNA-TPRα26 elongated dimer is fully conserved on the model of the RNA–G5C structure (Fig 5C). Notwithstanding, the large IDR region remains unstructured in both models, the tetramer and the dimer of G5C (Fig 5A and C), despite we observed that negatively charged residues (EE_1324–1325_) within the IDR determine the RNA binding affinity of RBS1 (Fig 2). Among other possibilities, this conformational flexible segment could relax protein rigidity required for RNA binding, as proposed for unconventional RBPs (52, 53).

Beyond the contribution of GEMIN5 domains for RNA binding affinity and oligomerization ability, other concurrent effects may contribute to the differential effects of GEMIN5 in gene expression regulation. Studies of how IDRs can mediate activator or repressor roles infer association with a repressive target (54), or regulation of the RNA-binding site (55). Notably, GEMIN5 recognizes diverse RNA targets and can regulate translation in a negative or a positive manner (26, 56, 57, 58, 59, 60) illustrating its functional diversity. Furthermore, the multiple functions of GEMIN5 are supported by the broad range of chase time observed in a temporal analysis of RNA–protein interactions through the mRNA life cycle (61).

In summary, our findings indicate that the presence of an extended RNA-binding surface constitutes a fundamental feature impacting on GEMIN5 functions. The mutational analysis shown in this study suggests that the G5C protein assembly involving the IDR facilitates RNA binding specificity. However, in the absence of the RBS region and, therefore, the loss of the pentamer structure, the protein TPRα26 is sufficient to promote RNA binding over the basal level of the TPR-like alone. We further show that specific modules within the C-terminal helical regions of GEMIN5 distinctly modulate RNA binding, the recruitment of the endogenous protein, and ribosome binding (Fig 6).

These data underscore a molecular basis for RNA regulation by the C-terminal region, ultimately providing insights into how pathological variants of this protein may impair distinct biological functions. Most of the clinical variants mapped in *Gemin5* gene hinder the dimerization module (31, 32). However, a few examples are reported in the RBS module (A1305Lfs, S1311L, H1364P, L1367P, A1410Q), mostly combined with another variant in the TPR-like module. Further pointing to the relevance of the two separate regions for GEMIN5 function, a recent article reported the presence of biallelic variant, carrying a S1144 frameshift (the last S residue of the ser-rich loop within the flexible loop between helix 19–20), and R1016C (a heterozygotic pathogenic mutant within the loop of helices 12-13 of the TPR domain) (29) in a patient with neurodevelopmental disorders (62). Interestingly, combination of structural data with functional assays indicated that adopting the tertiary structure is critical for G5C binding to cognate RNA ligands. Therefore, it is plausible that neurodevelopmental disorders mediated by *Gemin5* pathogenic mutations located on its C-terminal region are associated with abnormal RNA binding. Nevertheless, additional studies exploring the connections between the molecular mechanisms of GEMIN5 activities and the relation to pathogenic variant phenotypes will be enlightening.

## Materials and Methods

### Constructs

The plasmids pcDNA3-Xpress-G5_845–1508_ (G5C), pcDNA3-Xpress-G5_845–1436_ (TPRα28), pcDNA3-Xpress-G5_845–1286_ (TPRα26) (20), pET-G5_845–1286_ (TPRα26), pcDNA3-Xpress-G5_845–1097_ (TPR) (25), and pcDNA3-Xpress-G5_845–1508_-L1381D/M1384D/I1385D (G5C-Leu) (24) were previously reported. The plasmids pET-TPRα20 (845–1170) and pET-α18-α26 (1098–1286) were generated inserting the corresponding PCR product in pET28a via BamHI-NotI. Mutants pET-TPRα26 (EE_1132–1133_AA, E_1138_A, SSS_1141–1143_AAA, SY_1144–1145_AA, HW_1146–1148_AA, and E_1153_A), pcDNA3-Xpress-TPRα20, pcDNA3-Xpress-TPRα26-E_1138_A, pcDNA3-Xpress-TPRα26-SSS_1141–1143_AAA, pcDNA3-Xpress-TPRα26-SY_1144–1145_AA, and pET-RBS1 (21) mutants (EE_1324–1325_AA, EE_1347–1348_AA, D_1319_A, S_1322_A, T1_326_A, DE_1327–1329_AA, E_1329_A, and S_1331_A) were obtained by QuickChange mutagenesis. The primers used in each case are indicated in Table S1. All constructs were confirmed by DNA sequencing (Macrogen).

Table S1. Primer sequences used for GEMIN5 constructs.

### Expression and purification of proteins

*Escherichia coli* BL21 cells were transformed with plasmids encoding RBS1 proteins (RBS1-1412 (WT), RBS1-1361, RBS1-1326, EE_1324–1325_AA, EE_1347–1348_AA, D_1319_A, S_1322_A, T_1326_A, DE_1327–1329_AA, E_1329_A, and S_1331_A). Cultures were grown in LB medium at 37°C until an OD600 ∼0.6 was reached. Protein expression was induced with 0.5 mM Isopropyl β-D-1-thiogalactopyranoside (IPTG) for 2 h. Similarly, *E. coli* BL21 cells transformed with plasmids expressing TPRα20, α18–α26, TPRα26 WT, and TPRα26 mutants (EE_1132–1133_AA, E_1138_A, SSS_1141–1143_AAA, SY_1144–1145_AA, HW_1146–1148_AA, and E_1153_A), grown in LB medium at 37°C until reaching an OD600 of ∼0.6, were induced with 0.5 mM IPTG for 18 h at 20°C.

For protein purification, bacterial pellets were resuspended in binding buffer (20 mM NaH_2_PO_4_, 500 mM NaCl, 20 mM imidazole) and lysed by sonication. In the case of TPRα20, α18–α26, TPRα26 wild type, and the TPRα26 mutants, the binding buffer was supplemented with 8 M urea to preserve protein solubility. Cellular debris was removed twice by centrifugation at 16,000*g* for 30 min at 4°C. The clarified lysates were applied to His-GraviTrap columns (Healthcare), and recombinant proteins were eluted with 500 mM imidazole. Eluted proteins were dialyzed against phosphate buffer (pH 6.8) containing 1 mM DTT and stored at −20°C in 50% glycerol. Purified proteins were analyzed by SDS–polyacrylamide gel electrophoresis followed by Coomassie staining. Protein concentrations were determined by UV absorbance using extinction coefficients at 205 nm (63).

### RNA electrophoretic mobility shift assay

In vitro transcription of D5 and SL1 RNAs was performed as described (22, 64). RNA integrity was examined in 6% acrylamide 7 M urea denaturing gel electrophoresis. RNA–protein binding assays were performed in 10 μl of RNA-binding buffer (40 mM Tris–HCl, pH 7.5, 250 mM NaCl, 0.1% [wt/vol] βME) for 15 min at 4°C. Increasing concentrations of protein were incubated with a fixed amount of ^32^P-labeled RNA (<2 nM), prepared as a master mixture sufficient for all experimental points. Samples were resolved by electrophoresis on native 6.0% (29:1) polyacrylamide gels. Gels were run in TBE buffer (90 mM Tris–HCl, pH 8.4, 64.6 mM H_3_PO_3_, 2.5 mM EDTA) at 100 V at 4°C. Radiolabeled RNA and shifted complexes were visualized by autoradiography of dried gels. The proportion of shifted complexes was calculated relative to the free RNA probe run in parallel. Binding curves were generated using nonlinear regression with the one-site specific binding model in GraphPad Prism software (version 6.01).

### Protein expression in cultured cells

Human embryonic kidney HEK293 cells were cultured in Dulbeccoʼs Modified Eagle Medium (DMEM) supplemented with 5% fetal calf serum. Monolayers grown at 80% of confluency were transfected with the plasmid that expresses the Xpress-His constructs (G5C, G5C-leu, TPRα26, TPRα20, TPR, TPRα26 E_1138_A, TPRα26-SSS_1141–1143_AAA, and TPRα26-SY_1144–1145_AA) using Lipofectamine LTX (Thermo Fischer Scientific) and were harvested 24 h posttreatment (hpt) to prepare cell extracts with lysis buffer. The concentration of total protein was determined by the Bradford assay (BioRad). For SDS-resistant oligomer detection HEK293 cell lysates expressing the Xpress-GEMIN5 constructs (G5C, G5C-Leu, TPRα26, TPR, TPRα26 E_1138_A, TPRα26 SSS_1141–1143_AAA, and TPRα26 SY_1144–1145_AA) were mixed with SDS-loading buffer supplemented, or not, with 5 mM DTT, and heated 3 min at 92°C (40, 41, 42). Western blot analysis was carried out using anti-Xpress antibody.

### RNA binding assay in total cytoplasmic cell extracts

In vitro transcription of D5-1sm RNA was performed as described (41). Synthesized RNA was extracted by precipitation, its integrity confirmed in denatured gel electrophoresis and quantified by densitometry. For affinity purification of Xpress-GEMIN5 constructs associated with RNA, 100 μl of Dynabeads (Thermo Fisher Scientific) were first equilibrated in 5 mM Tris–HCl, pH 7.5, 1 M NaCl, and 0.5 mM EDTA. Beads were then washed with 0.1 M NaOH and 50 mM NaCl, followed by equilibration in 10 mM NaCl. Binding of D5-1sm RNA (20 pmol) to the beads was performed in refolding buffer (0.1 M HEPES, pH 7.4, 0.1 M NaCl, and 6 mM MgCl_2_) on a rotating wheel for 30 min at room temperature. Beads were collected using a magnet and washed three times. For the blocking step, D5-1sm beads were incubated in refolding buffer supplemented with BSA (final concentration 2 μg/μl) for 15 min (65). Beads were then collected and washed twice. Cytoplasmic extracts obtained from cells transfected with the indicated Xpress-GEMIN5 construct (G5C, TPRα28, TPRα26, TPRα20, TPR, TPRα26 E_1138_A, TPRα26 SSS_1141–1143_AAA, and TPRα26 SY_1144–1145_AA) (250 μg or 500 μg total protein to be in a linear range of the assay), prepared using lysis buffer (50 mM Tris–HCl, pH 7.8, 100 mM NaCl, 0.5% NP40, protease inhibitor [Roche]), were incubated with D5-1sm beads in Dyna buffer (0.1 M HEPES, pH 7.4, 0.1 M NaCl, and 0.45 mM MgCl_2_) for 30 min at room temperature. Beads were subsequently collected and washed three times. Incubation with higher amounts of cell extract resulted in saturating pull-down of the Xpress-tagged proteins. Pellets were denatured and separated by electrophoresis. The presence of Xpress-GEMIN5 proteins bound to the RNA-beads was analyzed by immunoblotting.

### Pull-down assays

HEK293 cells were co-transfected with the plasmid encoding G5_1–1508_-FLAG (66) together with the corresponding Xpress-His construct (TPRα26 WT, TPRα26 E_1138_A, TPRα26 SSS_1141–1143_AAA, and TPRα26 SY_1144–1145_AA). Cell extracts were prepared using lysis buffer containing 10 mM Tris–HCl, pH 7.5, 20 mM NaCl, 10 mM KCl, 5 mM MgCl_2_, 0.1% NP40, and protease inhibitors. Ni-NTA agarose resin (25 μl per pulldown) was equilibrated with lysis buffer lacking NP40 and subsequently incubated with the corresponding cell lysate (volume adjusted to 1 mg of total protein) for 1 h, at 4°C on a rotating wheel. After incubation, samples were centrifuged (16,000*g*, 4°C, 3 min) to remove the supernatant. The resin pellets were washed three times with washing buffer containing 10 mM Tris–HCl, pH 7.5, 20 mM NaCl, 10 mM KCl, 5 mM MgCl_2_, 10 mM imidazole, and protease inhibitors. Finally, pellets were resuspended in 30 μl of elution buffer (10 mM Tris–HCl, pH 7.5, 20 mM NaCl, 500 mM imidazole) and mixed with 20 μl of SDS-loading buffer, followed by heating at 92°C for 3 min. The presence of GEMIN5-FLAG associated with the Xpress-TPRα26 constructs was examined by immunoblotting.

### Subcellular fractionation

HEK293 cells, grown to 70–80% confluence, were transfected with the corresponding Xpress-His constructs. Cells were washed with ice-cold PBS and lysed in buffer 1 (15 mM Tris–HCl, pH 7.4, 80 mM KCl, 5 mM MgCl_2_, 1% Triton X-100, and protease inhibitors). Cellular debris was removed by centrifugation at 14,000*g* for 10 min at 4°C. The resulting supernatant (S30 fraction) was subjected to ultracentrifugation (391,000*g* for 1 h 30 min using a TLA100.2 rotor), generating the S100 fraction (supernatant) and the pellet containing native ribosomes (ribosomes together with associated factors). The ribosomal pellets were resuspended in 100 μl of buffer 1. Total protein levels in the S30 fraction were quantified by the Bradford assay, whereas ribosome concentration was estimated spectrophotometrically (14 A260 units = 1 mg/ml).

### Translation assays

siRNAs directed against *Gemin5* (CCU​UAA​UCA​AGA​AGA​GAA​AUU) or a control sequence (AUG​UAU​UGG​CCU​GUA​UUA​GUU) were obtained from Dharmacon. HEK293 cells at ∼70% confluence were transfected with 100 nM siRNA using Lipofectamine 2000 (Thermo Fisher Scientific) following the manufacturerʼs protocol.

For luciferase reporter assays, HEK293 cells treated or untreated with *Gemin5*-targeting siRNA were co-transfected with the plasmid expressing cap-dependent luciferase (pCAP-luc) (67) and the corresponding Xpress-His construct (TPRα26 WT, TPRα26 E_1138_A, TPRα26 SSS_1141–1143_AAA, and TPRα26 SY_1144–1145_AA). Cell lysates were prepared 24 h after transfection in 100 μl of lysis buffer (50 mM Tris–HCl, pH 7.8, 100 mM NaCl, 0.5% NP40). Protein concentrations were measured using the Bradford assay. Luciferase activity (RLU) per μg of total protein was calculated and normalized to the value obtained for the WT construct.

### Immunodetection

Equal amounts of total protein were separated by SDS–PAGE and transferred onto 0.2 μm PVDF membranes (Bio-Rad) using a semi-dry transfer system (Bio-Rad). Xpress-His-GEMIN5 proteins were detected using an anti-Xpress antibody (1:2,000) (Thermo Fisher Scientific), whereas GEMIN5-FLAG was identified with an anti-FLAG antibody (1:4,000) (Merck). Tubulin (1:4,000) (Merck) served as a loading control. An antibody against RACK1 (1:250) (Santa Cruz) was used as a ribosomal marker. HRP-conjugated secondary antibodies (Thermo Fisher Scientific) were applied according to the manufacturerʼs instructions.

### Statistical analyses

Statistical evaluation of the experimental data, including RNA binding assays, luciferase activity measurements, and densitometric quantification of bands from Western blot and EMSA analyses, were performed as follows. Each experiment was conducted independently at least three times. Data are presented as mean ± standard error of the mean (SEM). Statistical significance between two groups was determined using an unpaired two-tailed *t* test. Differences were considered statistically significant when *P* < 0.05. The corresponding *P*-values are indicated in the figures using asterisks as specified in the figure legends.

## Supplementary Material

Reviewer comments

## Data Availability

The data analyzed during this study are included in the article. Any underlying data with respect to the article will be made available upon reasonable request in the data availability statement.

## References

[1] Ray D, Laverty KU, Jolma A, Nie K, Samson R, Pour SE, Tam CL, von Krosigk N, Nabeel-Shah S, Albu M, (2023) RNA-binding proteins that lack canonical RNA-binding domains are rarely sequence-specific. Sci Rep 13: 5238. 10.1038/s41598-023-32245-9 PMC10066285 37002329

[2] Hentze MW, Sommerkamp P, Ravi V, Gebauer F (2025) Rethinking RNA-binding proteins: Riboregulation challenges prevailing views. Cell 188: 4811–4827. 10.1016/j.cell.2025.06.021 40912239

[3] Agarwal A, Bahadur RP (2023) Modular architecture and functional annotation of human RNA-binding proteins containing RNA recognition motif. Biochimie 209: 116–130. 10.1016/j.biochi.2023.01.017 36716848

[4] Dominguez D, Freese P, Alexis MS, Su A, Hochman M, Palden T, Bazile C, Lambert NJ, Van Nostrand EL, Pratt GA, (2018) Sequence, structure, and context preferences of human RNA binding proteins. Mol Cell 70: 854–867.e9. 10.1016/j.molcel.2018.05.001 PMC6062212 29883606

[5] Helder S, Blythe AJ, Bond CS, Mackay JP (2016) Determinants of affinity and specificity in RNA-binding proteins. Curr Opin Struct Biol 38: 83–91. 10.1016/j.sbi.2016.05.005 27315040

[6] Zeke A, Schád É, Horváth T, Abukhairan R, Szabó B, Tantos A (2022) Deep structural insights into RNA-binding disordered protein regions. Wiley Interdiscip Rev RNA 13: e1714. 10.1002/wrna.1714 PMC9539567 35098694

[7] Protter DSW, Rao BS, Van Treeck B, Lin Y, Mizoue L, Rosen MK, Parker R (2018) Intrinsically disordered regions can contribute promiscuous interactions to RNP granule assembly. Cell Rep 22: 1401–1412. 10.1016/j.celrep.2018.01.036 PMC5824733 29425497

[8] Ottoz DSM, Berchowitz LE (2020) The role of disorder in RNA binding affinity and specificity. Open Biol 10: 200328. 10.1098/rsob.200328 PMC7776568 33352065

[9] Bigman LS, Iwahara J, Levy Y (2022) Negatively charged disordered regions are prevalent and functionally important across proteomes. J Mol Biol 434: 167660. 10.1016/j.jmb.2022.167660 35659505

[10] Loughlin FE, Lukavsky PJ, Kazeeva T, Reber S, Hock EM, Colombo M, Von Schroetter C, Pauli P, Cléry A, Muhlemann O, (2019) The solution structure of FUS bound to RNA reveals a bipartite mode of RNA recognition with both sequence and shape specificity. Mol Cell 73: 490–504.e6. 10.1016/j.molcel.2018.11.012 30581145

[11] Fenton M, Gregory E, Daughdrill G (2023) Protein disorder and autoinhibition: The role of multivalency and effective concentration. Curr Opin Struct Biol 83: 102705. 10.1016/j.sbi.2023.102705 PMC10841074 37778184

[12] Zaharias S, Fargason T, Greer R, Song Y, Zhang J (2023) Electronegative clusters modulate folding status and RNA binding of unstructured RNA-binding proteins. Protein Sci 32: e4643. 10.1002/pro.4643 PMC10127258 37060324

[13] Qiu C, Zhang Z, Wine RN, Campbell ZT, Zhang J, Hall TMT (2023) Intra- and inter-molecular regulation by intrinsically-disordered regions governs PUF protein RNA binding. Nat Commun 14: 7323. 10.1038/s41467-023-43098-1 PMC10641069 37953271

[14] Wang X, Levy Y, Iwahara J (2025) Competition between nucleic acids and intrinsically disordered regions within proteins. Acc Chem Res 58: 2415–2424. 10.1021/acs.accounts.5c00261 PMC12369953 40631955

[15] Yang J, Gao M, Xiong J, Su Z, Huang Y (2019) Features of molecular recognition of intrinsically disordered proteins via coupled folding and binding. Protein Sci 28: 1952–1965. 10.1002/pro.3718 PMC6798136 31441158

[16] Battle DJ, Lau CK, Wan L, Deng H, Lotti F, Dreyfuss G (2006) The Gemin5 protein of the SMN complex identifies snRNAs. Mol Cell 23: 273–279. 10.1016/j.molcel.2006.05.036 16857593

[17] Lau CK, Bachorik JL, Dreyfuss G (2009) Gemin5-snRNA interaction reveals an RNA binding function for WD repeat domains. Nat Struct Mol Biol 16: 486–491. 10.1038/nsmb.1584 19377484

[18] Xu C, Ishikawa H, Izumikawa K, Li L, He H, Nobe Y, Yamauchi Y, Shahjee HM, Wu X-H, Yu Y-T, (2016) Structural insights into Gemin5-guided selection of pre-snRNAs for snRNP assembly. Genes Dev 30: 2376–2390. 10.1101/gad.288340.116 PMC5131778 27881600

[19] Jin W, Wang Y, Liu CP, Yang N, Jin M, Cong Y, Wang M, Xu RM (2016) Structural basis for snRNA recognition by the double-WD40 repeat domain of Gemin5. Genes Dev 30: 2391–2403. 10.1101/gad.291377.116 PMC5131779 27881601

[20] Moreno-Morcillo M, Francisco-Velilla R, Embarc-Buh A, Fernández-Chamorro J, Ramón-Maiques S, Martinez-Salas E (2020) Structural basis for the dimerization of Gemin5 and its role in protein recruitment and translation control. Nucleic Acids Res 48: 788–801. 10.1093/nar/gkz1126 PMC6954437 31799608

[21] Fernandez-Chamorro J, Piñeiro D, Gordon JM, Ramajo J, Francisco-Velilla R, Macias MJ, Martinez-Salas E (2014) Identification of novel non-canonical RNA-binding sites in Gemin5 involved in internal initiation of translation. Nucleic Acids Res 42: 5742–5754. 10.1093/nar/gku177 PMC4027194 24598255

[22] Francisco-Velilla R, Embarc-Buh A, Rangel-Guerrero S, Basu S, Kundu S, Martinez-Salas E (2020) RNA-protein coevolution study of Gemin5 uncovers the role of the PXSS motif of RBS1 domain for RNA binding. RNA Biol 17: 1331–1341. 10.1080/15476286.2020.1762054 PMC7549667 32476560

[23] Embarc-Buh A, Francisco-Velilla R, Camero S, Pérez-Cañadillas JM, Martínez-Salas E (2021) The RBS1 domain of Gemin5 is intrinsically unstructured and interacts with RNA through conserved Arg and aromatic residues. RNA Biol 18: 496–506. 10.1080/15476286.2021.1962666 PMC8677033 34424823

[24] Guo Q, Zhao S, Francisco-Velilla R, Zhang J, Embarc-Buh A, Abellan S, Lv M, Tang P, Gong Q, Shen H, (2022) Structural basis for Gemin5 decamer-mediated mRNA binding. Nat Commun 13: 5166. 10.1038/s41467-022-32883-z PMC9440017 36056043

[25] Francisco-Velilla R, Abellan S, Embarc-Buh A, Martinez-Salas E (2024) Oligomerization regulates the interaction of Gemin5 with members of the SMN complex and the translation machinery. Cell Death Discov 10: 306. 10.1038/s41420-024-02057-5 PMC11213948 38942768

[26] Francisco-Velilla R, Fernandez-Chamorro J, Ramajo J, Martinez-Salas E (2016) The RNA-binding protein Gemin5 binds directly to the ribosome and regulates global translation. Nucleic Acids Res 44: 8335–8351. 10.1093/nar/gkw702 PMC5041490 27507887

[27] Kour S, Rajan DS, Fortuna TR, Anderson EN, Ward C, Lee Y, Lee S, Shin YB, Chae JH, Choi M, (2021) Loss of function mutations in GEMIN5 cause a neurodevelopmental disorder. Nat Commun 12: 2558. 10.1038/s41467-021-22627-w PMC8105379 33963192

[28] Saida K, Tamaoki J, Sasaki M, Haniffa M, Koshimizu E, Sengoku T, Maeda H, Kikuchi M, Yokoyama H, Sakamoto M, (2021) Pathogenic variants in the survival of motor neurons complex gene GEMIN5 cause cerebellar atrophy. Clin Genet 100: 722–730. 10.1111/cge.14066 34569062

[29] Francisco-Velilla R, Embarc-Buh A, Del Caño-Ochoa F, Abellan S, Vilar M, Alvarez S, Fernandez-Jaen A, Kour S, Rajan DS, Pandey UB, (2022) Functional and structural deficiencies of Gemin5 variants associated with neurological disorders. Life Sci Alliance 5: e202201403. 10.26508/lsa.202201403 PMC8989681 35393353

[30] Rajan DS, Kour S, Fortuna TR, Cousin MA, Barnett SS, Niu Z, Babovic-Vuksanovic D, Klee EW, Kirmse B, Innes M, (2022) Autosomal recessive cerebellar atrophy and spastic ataxia in patients with pathogenic biallelic variants in *GEMIN5*. Front Cell Dev Biol 10: 783762. 10.3389/fcell.2022.783762 PMC8918504 35295849

[31] Nelson CH, Pandey UB (2024) Function and dysfunction of GEMIN5: Understanding a novel neurodevelopmental disorder. Neural Regen Res 19: 2377–2386. 10.4103/NRR.NRR-D-23-01614 PMC11090446 38526274

[32] Martinez-Salas E, Francisco-Velilla R (2026) GEMIN5 and neurodevelopmental diseases: From functional insights to disease perception. Neural Regen Res 21: 187–194. 10.4103/NRR.NRR-D-24-01010 PMC12094563 39819844

[33] Zhang X, Guo Y, Xu L, Wang Y, Sheng G, Gao F, Yuan Z (2023) Novel compound heterozygous mutation and phenotype in the tetratricopeptide repeat-like domain of the GEMIN5 gene in two Chinese families. J Hum Genet 68: 789–792. 10.1038/s10038-023-01184-8 37479787

[34] Ibrahim N, Naz S, Mattioli F, Guex N, Sharif S, Iqbal A, Ansar M, Reymond A (2023) A biallelic truncating variant in the TPR domain of GEMIN5 associated with intellectual disability and cerebral atrophy. Genes (Basel) 14: 707. 10.3390/genes14030707 PMC10048441 36980979

[35] Cascajo-Almenara MV, Juliá-Palacios N, Urreizti R, Sánchez-Cuesta A, Fernández-Ayala DM, García-Díaz E, Oliva C, O Callaghan MDM, Paredes-Fuentes AJ, Moreno-Lozano PJ, (2024) Mutations of GEMIN5 are associated with coenzyme Q_10_ deficiency: Long-term follow-up after treatment. Eur J Hum Genet 32: 426–434. 10.1038/s41431-023-01526-2 PMC10999423 38316953

[36] Zhang J, Liu X, Zhu G, Wan L, Liang Y, Li N, Huang M, Yang G (2024) Expanding the clinical phenotype and genetic spectrum of GEMIN5 disorders: Early-infantile developmental and epileptic encephalopathies. Brain Behav 14: e3535. 10.1002/brb3.3535 PMC11109505 38773790

[37] Martinez-Salas E, Abellan S, Francisco-Velilla R (2025) Understanding GEMIN5 interactions: From structural and functional insights to selective translation. Wiley Interdiscip Rev RNA 16: e70008. 10.1002/wrna.70008 PMC11965781 40176294

[38] Francisco-Velilla R, Fernandez-Chamorro J, Dotu I, Martinez-Salas E (2018) The landscape of the non-canonical RNA-binding site of Gemin5 unveils a feedback loop counteracting the negative effect on translation. Nucleic Acids Res 46: 7339–7353. 10.1093/nar/gky361 PMC6101553 29771365

[39] Hu G, Katuwawala A, Wang K, Wu Z, Ghadermarzi S, Gao J, Kurgan L (2021) flDPnn: Accurate intrinsic disorder prediction with putative propensities of disorder functions. Nat Commun 12: 4438. 10.1038/s41467-021-24773-7 PMC8295265 34290238

[40] Wang K, Hu G, Basu S, Kurgan L (2026) flDPnn3: Fast and accurate prediction of intrinsic disorder in protein sequences. J Mol Biol 438: 169629. 10.1016/j.jmb.2026.169629 41500381

[41] Abellan S, Escos A, Francisco-Velilla R, Martinez-Salas E (2025) Impact of Gemin5 in protein synthesis: Phosphoresidues of the dimerization domain regulate ribosome binding. RNA Biol 22: 1–15. 10.1080/15476286.2025.2540654 PMC12323416 40734649

[42] Pacheco A, López de Quinto S, Ramajo J, Fernández N, Martínez-Salas E (2009) A novel role for Gemin5 in mRNA translation. Nucleic Acids Res 37: 582–590. 10.1093/nar/gkn979 PMC2632916 19066202

[43] Ponchon L, Dardel F (2007) Recombinant RNA technology: The tRNA scaffold. Nat Methods 4: 571–576. 10.1038/nmeth1058 17558412

[44] Fernandez-Chamorro J, Francisco-Velilla R, Ramajo J, Martinez-Salas E (2019) Rab1b and ARF5 are novel RNA-binding proteins involved in FMDV IRES-driven RNA localization. Life Sci Alliance 2: e201800131. 10.26508/lsa.201800131 PMC6337736 30655362

[45] Lou X, Ma B, Zhuang Y, Xiao X, Minze LJ, Xing J, Zhang Z, Li XC. 2022. Structural studies of the coiled-coil domain of TRIM75 reveal a tetramer architecture facilitating its E3 ligase complex. Comput Struct Biotechnol J 20:4921–4929, 10.1016/j.csbj.2022.08.069 PMC9471973 36147661

[46] Wang B, Wang Y, Pan T, Zhou L, Ran Y, Zou J, Yan X, Wen Z, Lin S, Ren A, (2025) Targeting a key disulfide linkage to regulate RIG-I condensation and cytosolic RNA-sensing. Nat Cell Biol 27: 817–834. 10.1038/s41556-025-01646-5 40229436

[47] Rasmussen HØ, Nielsen J, de Poli A, Otzen DE, Pedersen JS (2023) Tau fibrillation induced by heparin or a lysophospholipid show different initial oligomer formation. J Mol Biol 435: 168194. 10.1016/j.jmb.2023.168194 37437877

[48] Meng EC, Goddard TD, Pettersen EF, Couch GS, Pearson ZJ, Morris JH, Ferrin TE (2023) UCSF ChimeraX: Tools for structure building and analysis. Protein Sci 32: e4792. 10.1002/pro.4792 PMC10588335 37774136

[49] Neuenkirchen N, Englbrecht C, Ohmer J, Ziegenhals T, Chari A, Fischer U (2015) Reconstitution of the human U snRNP assembly machinery reveals stepwise Sm protein organization. EMBO J 34: 1925–1941. 10.15252/embj.201490350 PMC4547896 26069323

[50] Jumper J, Evans R, Pritzel A, Green T, Figurnov M, Ronneberger O, Tunyasuvunakool K, Bates R, Žídek A, Potapenko A, (2021) Highly accurate protein structure prediction with AlphaFold. Nature 596: 583–589. 10.1038/s41586-021-03819-2 PMC8371605 34265844

[51] Varadi M, Anyango S, Deshpande M, Nair S, Natassia C, Yordanova G, Yuan D, Stroe O, Wood G, Laydon A, (2022) AlphaFold protein structure database: Massively expanding the structural coverage of protein-sequence space with high-accuracy models. Nucleic Acids Res 50: D439–D444. 10.1093/nar/gkab1061 PMC8728224 34791371

[52] Chatterjee S, Maity A, Bahadur RP (2025) Conformational switches in human RNA binding proteins involved in neurodegeneration. Biochim Biophys Acta Gen Subj 1869: 130760. 10.1016/j.bbagen.2025.130760 39798673

[53] Kucerikova M, Bonczek O, Olivares-Illana V, Rodriguez-Rodriguez A, Sampedro JG, Hernychova L, Hrabal V, Zatloukalova P, Krejcir R, Fahraeus R, (2025) Reduced structural rigidity of MDMX protein enhances binding to TP53 mRNA. Biosci Rep 45: 683–696. 10.1042/BSR20253646 PMC12751038 41230869

[54] Khan MR, Li L, Pérez-Sánchez C, Saraf A, Florens L, Slaughter BD, Unruh JR, Si K (2015) Amyloidogenic oligomerization transforms *Drosophila* Orb2 from a translation repressor to an activator. Cell 163: 1468–1483. 10.1016/j.cell.2015.11.020 PMC4674814 26638074

[55] Iserman C, Desroches Altamirano C, Jegers C, Friedrich U, Zarin T, Fritsch AW, Mittasch M, Domingues A, Hersemann L, Jahnel M, (2020) Condensation of Ded1p promotes a translational switch from housekeeping to stress protein production. Cell 181: 818–831.e19. 10.1016/j.cell.2020.04.009 PMC7237889 32359423

[56] Piñeiro D, Ramajo J, Bradrick SS, Martínez-Salas E (2012) Gemin5 proteolysis reveals a novel motif to identify L protease targets. Nucleic Acids Res 40: 4942–4953. 10.1093/nar/gks172 PMC3367203 22362733

[57] Workman E, Kalda C, Patel A, Battle DJ (2015) Gemin5 binds to the survival motor neuron mRNA to regulate SMN expression. J Biol Chem 290: 15662–15669. 10.1074/jbc.M115.646257 PMC4505476 25911097

[58] Garcia-Moreno M, Noerenberg M, Ni S, Järvelin AI, González-Almela E, Lenz CE, Bach-Pages M, Cox V, Avolio R, Davis T, (2019) System-wide profiling of RNA-binding proteins uncovers key regulators of virus infection. Mol Cell 74: 196–211.e11. 10.1016/j.molcel.2019.01.017 PMC6458987 30799147

[59] Van Nostrand EL, Pratt GA, Yee BA, Wheeler EC, Blue SM, Mueller J, Park SS, Garcia KE, Gelboin-Burkhart C, Nguyen TB, (2020) Principles of RNA processing from analysis of enhanced CLIP maps for 150 RNA binding proteins. Genome Biol 21: 90. 10.1186/s13059-020-01982-9 PMC7137325 32252787

[60] Embarc-Buh A, Francisco-Velilla R, Garcia-Martin JA, Abellan S, Ramajo J, Martinez-Salas E (2022) Gemin5-dependent RNA association with polysomes enables selective translation of ribosomal and histone mRNAs. Cell Mol Life Sci 79: 490. 10.1007/s00018-022-04519-4 PMC9392717 35987821

[61] Choi Y, Um B, Na Y, Kim J, Kim JS, Kim VN (2024) Time-resolved profiling of RNA binding proteins throughout the mRNA life cycle. Mol Cell 84: 1764–1782.e10. 10.1016/j.molcel.2024.03.012 38593806

[62] Thaw M, Thida Nwe H, Thura K, Wynn KKK, Mohammad A, Grote H (2026) Advanced presentation of GEMIN5 neurodevelopmental disorder in an elderly female. Cureus 18: e115273. 10.7759/cureus.115273 PMC13615410 42800983

[63] Anthis NJ, Clore GM (2013) Sequence-specific determination of protein and peptide concentrations by absorbance at 205 nm. Protein Sci 22: 851–858. 10.1002/pro.2253 PMC3690723 23526461

[64] López de Quinto S, Lafuente E, Martínez-Salas E (2001) IRES interaction with translation initiation factors: Functional characterization of novel RNA contacts with eIF3, eIF4B, and eIF4GII. RNA 7: 1213–1226. 10.1017/s1355838201010433 PMC1370167 11565745

[65] Fernandez-Chamorro J, Francisco-Velilla R, Embarc-Buh A, Martinez-Salas E (2021) Identification of RNA-binding proteins associated to RNA structural elements. Methods Mol Biol 2323: 109–119. 10.1007/978-1-0716-1499-0_9 34086277

[66] Piñeiro D, Fernández N, Ramajo J, Martínez-Salas E (2013) Gemin5 promotes IRES interaction and translation control through its C-terminal region. Nucleic Acids Res 41: 1017–1028. 10.1093/nar/gks1212 PMC3553989 23221641

[67] Lozano G, Francisco-Velilla R, Martinez-Salas E (2018) Ribosome-dependent conformational flexibility changes and RNA dynamics of IRES domains revealed by differential SHAPE. Sci Rep 8: 5545. 10.1038/s41598-018-23845-x PMC5882922 29615727

