# Supplementary material for: Contribution of disordered regions of the essential protein GEMIN5 to RNA binding
Source: Life Sci Alliance. 2026 Oct 27;10(1):e202603860. doi: 10.26508/lsa.202603860 (PMC13714362; doi:10.26508/lsa.202603860)
Supplement: Supplementary file 2 [file LSA-2026-03860_SdataF2.pdf]

Figure 2

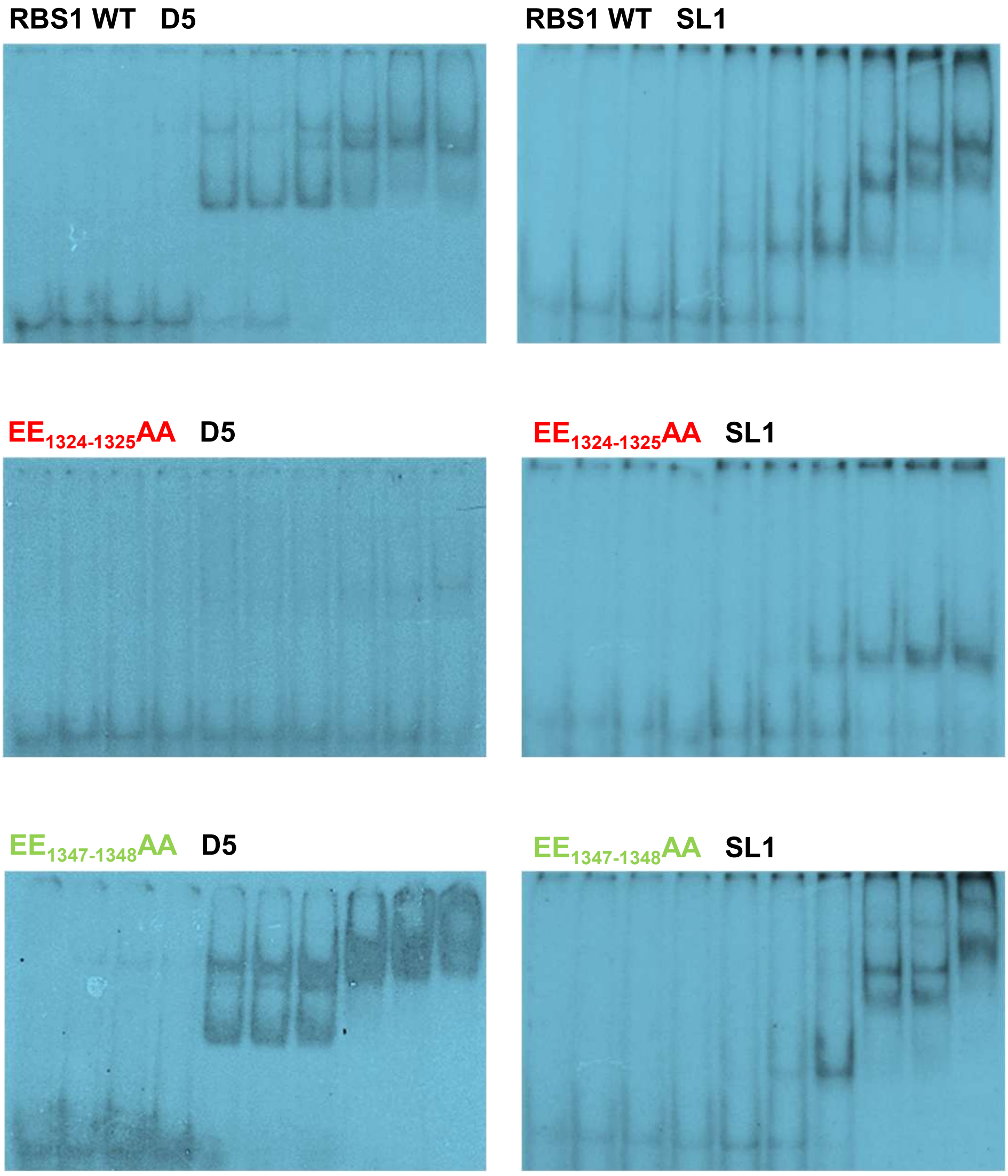
RBS1 WT D5
RBS1 WT SL1
EE1324-1325AA D5
EE1324-1325AA SL1
EE1347-1348AA D5
EE1347-1348AA SL1
