# Supplementary material for: Contribution of disordered regions of the essential protein GEMIN5 to RNA binding
Source: Life Sci Alliance. 2026 Oct 27;10(1):e202603860. doi: 10.26508/lsa.202603860 (PMC13714362; doi:10.26508/lsa.202603860)
Supplement: Supplementary file 3 [file LSA-2026-03860_SdataF3.pdf]

Figure 3C

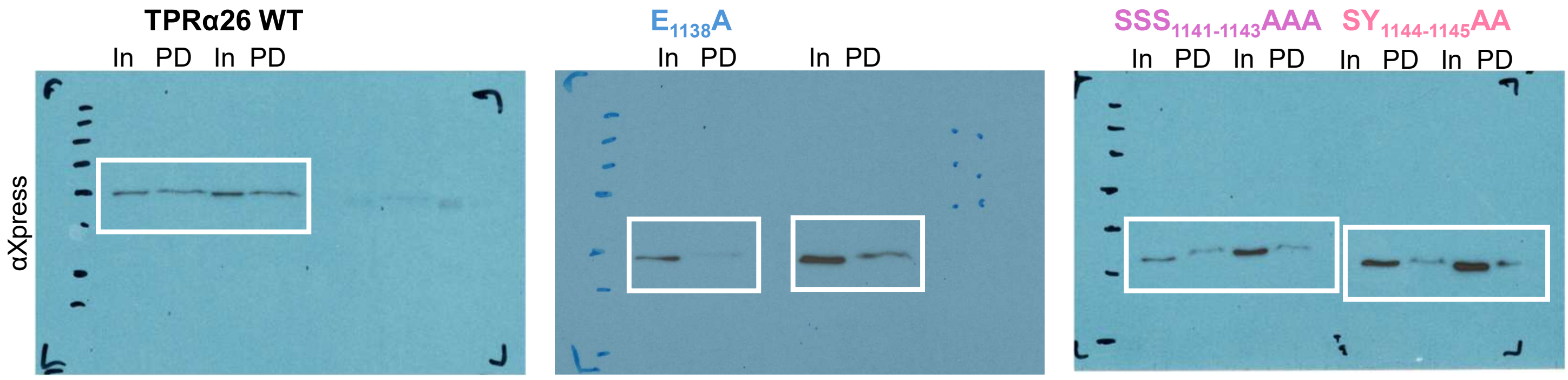

TPRα26 WT
In PD In PD
E1138A
In PD In PD
SSS1141-1143AAA
SY1144-1145AA
In PD In PD In PD In PD
αXpress
