# Supplementary material for: Contribution of disordered regions of the essential protein GEMIN5 to RNA binding
Source: Life Sci Alliance. 2026 Oct 27;10(1):e202603860. doi: 10.26508/lsa.202603860 (PMC13714362; doi:10.26508/lsa.202603860)
Supplement: Supplementary file 4 [file LSA-2026-03860_SdataF4.pdf]

Figure 4

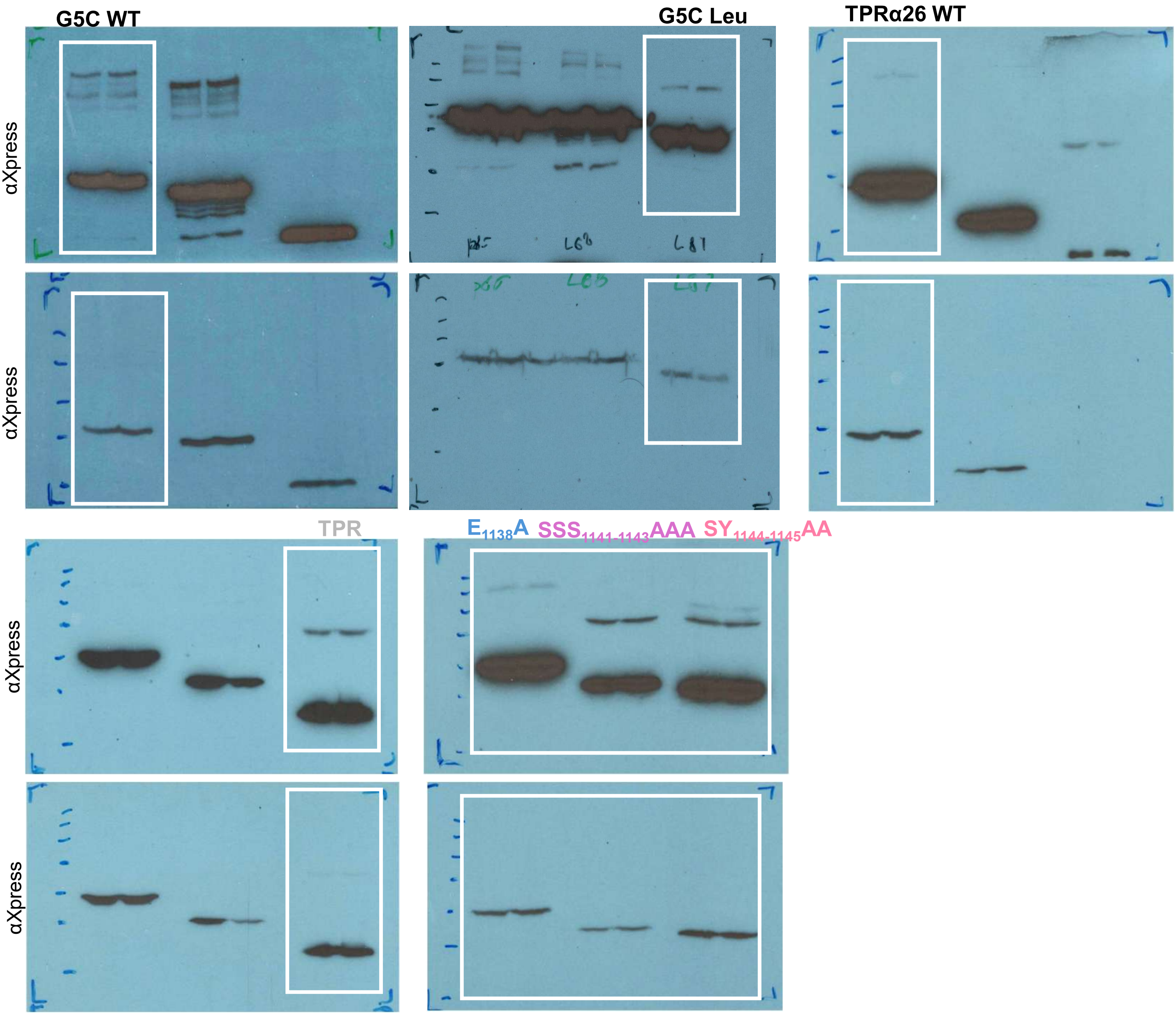

G5C WT
G5C Leu
TPRα26 WT
αXpress
αXpress
TPR
E1138A
SSS1141-1143AAA
SY1144-1145AA
αXpress
αXpress
