# Supplementary material for: Contribution of disordered regions of the essential protein GEMIN5 to RNA binding
Source: Life Sci Alliance. 2026 Oct 27;10(1):e202603860. doi: 10.26508/lsa.202603860 (PMC13714362; doi:10.26508/lsa.202603860)

Figure 6B

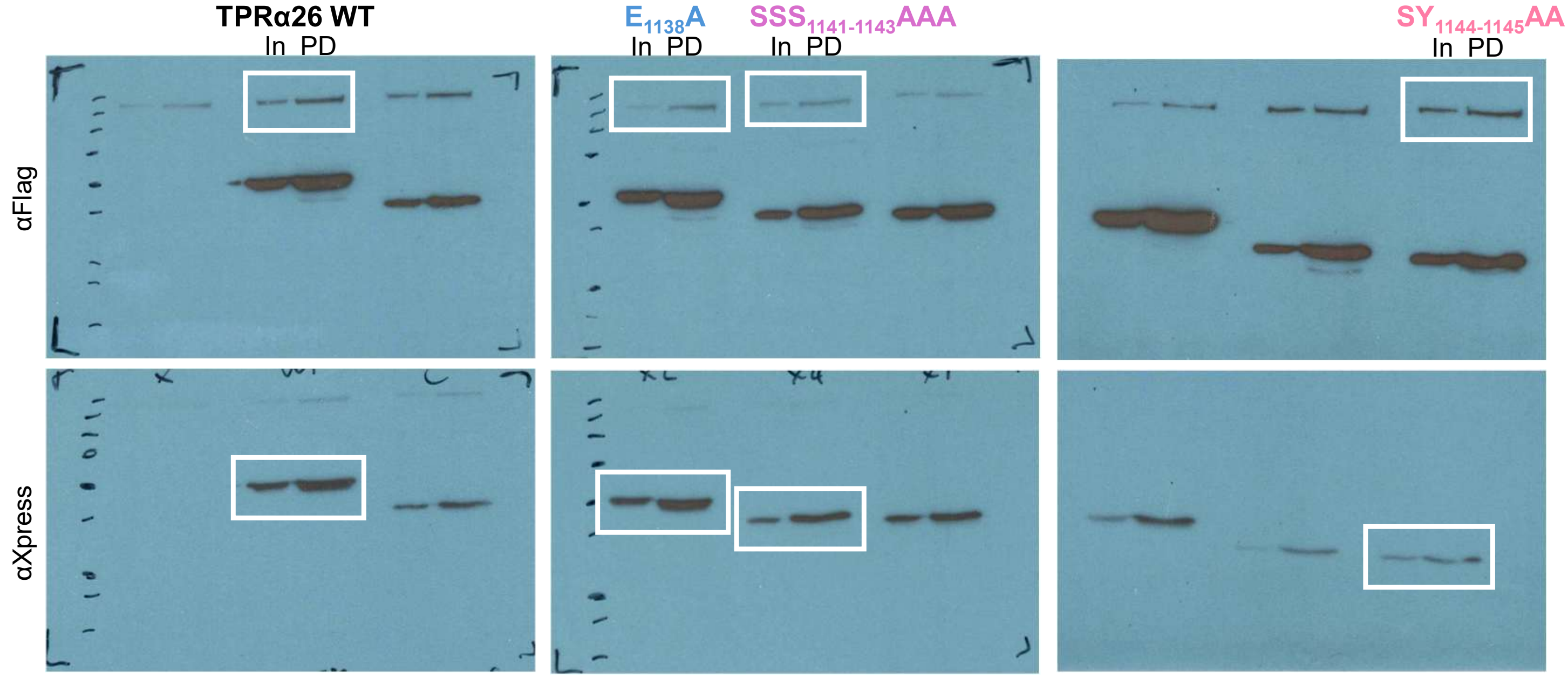

Figure 6D

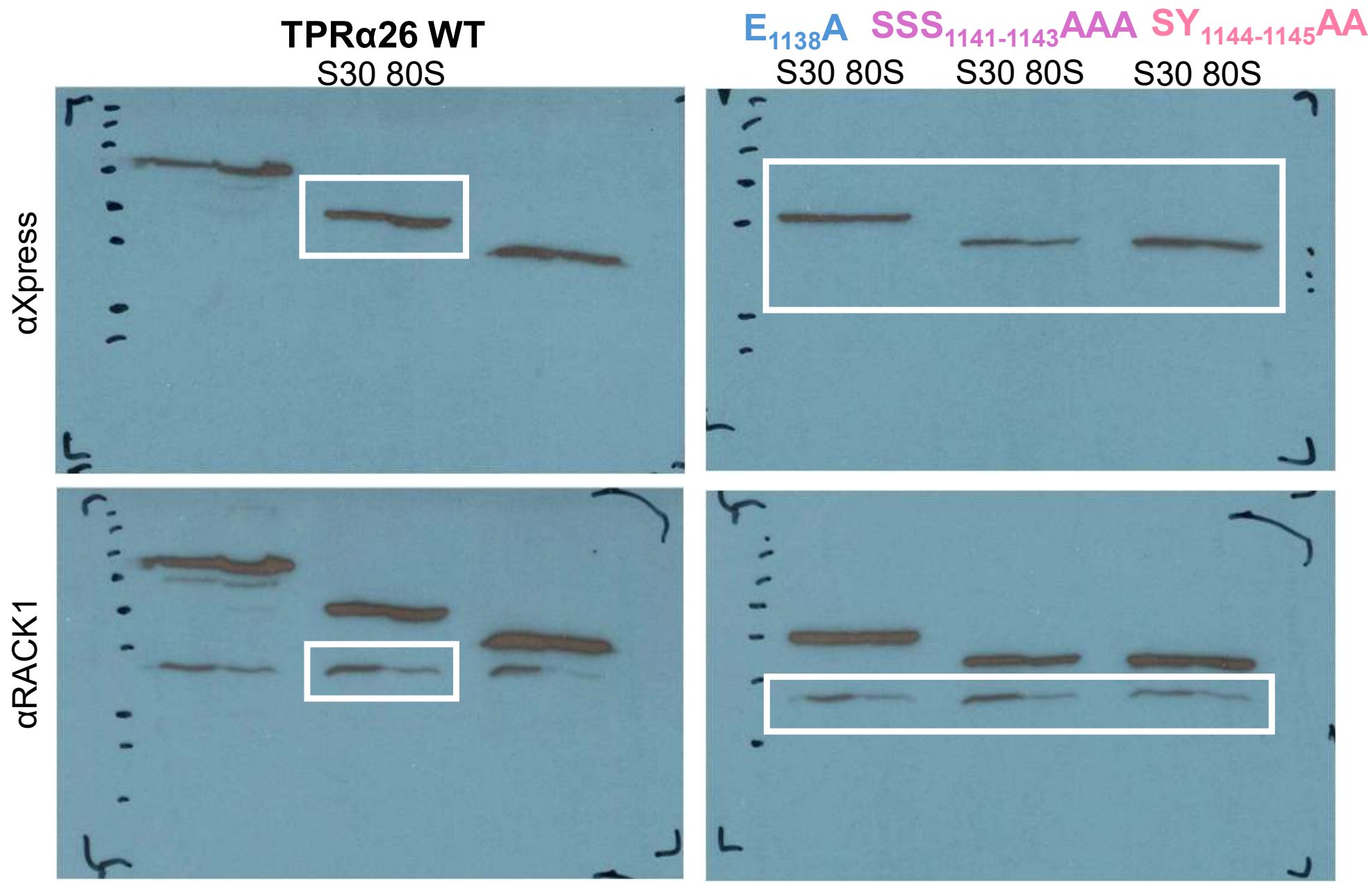

Supplement: Supplementary file 5 [file LSA-2026-03860_SdataF6.pdf]
