# Supplementary material for: Contribution of disordered regions of the essential protein GEMIN5 to RNA binding
Source: Life Sci Alliance. 2026 Oct 27;10(1):e202603860. doi: 10.26508/lsa.202603860 (PMC13714362; doi:10.26508/lsa.202603860)
Supplement: Supplementary file 6 [file LSA-2026-03860_review_history.pdf]

Contribution of disordered regions of the essential protein GEMIN5 to RNA binding

Rosario Francisco-Velilla, Azadeh Nahavandi Araghi, Jorge Ramajo, Salvador Abellan, and Encarnacion Martinez-Salas
DOI: https://doi.org/10.26508/lsa.202603860

Corresponding author(s): Encarnacion Martinez-Salas, Centro de Biologia Molecular Severo Ochoa, CSIC-UAM

Review Timeline:

Submission Date:	2026-07-27
Editorial Decision:	2026-09-09
Revision Received:	2026-09-28
Editorial Decision:	2026-09-29
Revision Received:	2026-10-05
Accepted:	2026-10-07

Scientific Editor: Tim Fessenden

Transaction Report:

(Note: With the exception of the correction of typographical or spelling errors that could be a source of ambiguity, letters and reports are not edited. The original formatting of letters and referee reports may not be reflected in this compilation.)

September 9, 2026

Re: Life Science Alliance manuscript #LSA-2026-03860

Prof. Encarnacion Martinez-Salas
Centro de Biologia Molecular Severo Ochoa, CSIC-UAM
Genome Dynamics and Function
Nicolas Cabrera, 1, Cantoblanco
Madrid 28049
Spain

Dear Dr. Martinez-Salas,

Thank you for submitting your manuscript entitled "Contribution of disordered regions of the essential protein GEMIN5 to RNA binding" to Life Science Alliance. The manuscript was assessed by expert reviewers, whose comments are appended to this letter.

As you will see, reviewers were unanimous in their support for this work with overall few critiques. Reviewer 2 requested additional RNA binding assays which we concur may strengthen these results but we leave this as optional. We encourage you to add estimated Kd values where appropriate, as requested by this reviewer. Finally we agree with Reviewers 1 and 3 that structural predictions should be carefully framed as preliminary. We look forward to a revised manuscript with the above changes, which we will evaluate without further input from reviewers.

I would be happy to discuss the revision in more detail via email or phone/videoconferencing. Please let me know which option you prefer, if any.

To upload the revised version of your manuscript, please log in to your account: https://lsa.msubmit.net/cgi-bin/main.plex

You will be guided to complete the submission of your revised manuscript and to fill in all necessary information. Please get in touch in case you do not know or remember your login name.

The typical timeframe for revisions is three months. Please note that papers are generally considered through only one revision cycle, so strong support from the referees on the revised version is needed for acceptance.

While you are revising your manuscript, please also attend to the below editorial points to help expedite the publication of your manuscript. Please direct any editorial questions to the journal office. When submitting the revision, please include a letter addressing the reviewers' comments point by point.

Thank you for this interesting contribution to Life Science Alliance. We hope that the comments below will prove constructive as your work progresses, and we are looking forward to receiving your revised manuscript.

Sincerely,

Tim Fessenden
Scientific Editor
Life Science Alliance

A. THESE ITEMS ARE REQUIRED FOR REVISIONS

-- A letter addressing the reviewers' comments point by point.

-- An editable version of the final text (.DOC or .DOCX) is needed for copyediting (no PDFs).

-- High-resolution figure, supplementary figure and video files uploaded as individual files: See our detailed guidelines for preparing your production-ready images, https://www.life-science-alliance.org/authors

-- Summary blurb (enter in submission system): A short text summarizing in a single sentence the study (max. 200 characters including spaces). This text is used in conjunction with the titles of papers, hence should be informative and complementary to

the title and running title. It should describe the context and significance of the findings for a general readership; it should be written in the present tense and refer to the work in the third person. Author names should not be mentioned.

-- By submitting a revision, you attest that you are aware of our payment policies found here: https://www.life-science-alliance.org/copyright-license-fee

B. MANUSCRIPT ORGANIZATION AND FORMATTING:

Full guidelines are available on our Instructions for Authors page, https://www.life-science-alliance.org/authors

We encourage our authors to provide original source data, particularly uncropped/-processed electrophoretic blots and spreadsheets for the main figures of the manuscript. If you would like to add source data, we would welcome one PDF/Excel-file per figure for this information. These files will be linked as supplementary "Source Data" files.

IMPORTANT: It is Life Science Alliance policy that if requested, original data images must be made available. Failure to provide original images upon request will result in unavoidable delays in publication. Please ensure that you have access to all original microscopy and blot data images before submitting your revision.

Reviewer #1 (Comments to the Authors (Required)):

The manuscript by Francisco-Velilla, R., et al. presents the biochemical, structural, and functional characterisation of the RNA-binding domain of GEMIN5. The authors identify two key uncharacterized regions that together constitute an extended RNA-binding surface. Using truncation mutants, site-directed mutagenesis, in vitro EMSAs, cellular pulldown assay, and AlphaFold modelling, the study convincingly links the structural features to RNA binding, self-dimerisation, and ribosome association. Overall, this is a well-structured and cohesive study that I recommend for publication after the authors address a few minor points in the text.
Minor points:
Briefly elaborate on the structural rationale for why extending the TPR domain to TPRα20/26 restores RNA binding.
Ensure figure legends clearly state sample sizes/replicates for the oligomerisation Western blots
(Fig. 4).
Keep the AF modelling explicitly framed as a predictive structural hypothesis throughout.

Reviewer #2 (Comments to the Authors (Required)):

GEMIN5, as the RNA-binding protein of the SMN complex linked to human disease, comprises an N-terminal WD40 repeat domain and a C-terminal region (G5C) that includes a tetratricopeptide repeat (TPR)-like dimerization module, a non-canonical RNA-binding site (RBS), and an intrinsically disordered region (IDR), but the potential role of the IDR remains obscure. In this manuscript, Rosario et al. found the RNA-binding domain of GEMIN5 consists of an extended binding surface comprising a serine-rich loop and an acidic cluster within the IDR and the specific modules within the C-terminal helical region modulate RNA-binding, recruitment of the endogenous protein, ribosome binding. Overall, these findings are potentially interesting and most of the experiments are well done and support their conclusions. Following comments may help for further improvement.

1.In Figure 1A, the start and end amino acid residues of all truncated constructs should be mapped according to the full-length protein sequence.
2.In Figure 1E, RNA-binding in vitro of TPRα28 and G5C full-length protein should be added.
3.All data pertaining to RNA-binding capacity should include calculated Kd values and statistical significance analyses of Kd to demonstrate the differences between the wild-type and mutant proteins.

Reviewer #3 (Comments to the Authors (Required)):

The manuscript by Francisco-Velilla and colleagues investigate the mechanisms governing RNA binding in GEMIN5, an essential RNA-binding protein (RBP) linked to neurodevelopmental disorders. Using a combination of biochemical assays, cell culture truncations/mutations, and computational modeling, the authors identify an extended RNA-binding surface located in GEMIN5's C-terminal domain (G5C). Specifically, they demonstrate that RNA binding is regulated by two distinct unstructured regions: a serine-rich loop within an interhelical region and an acidic cluster within an intrinsically disordered region. The study reveals that while mutations in either region impair RNA binding, the TPR-extended protein moiety remains sufficient to bind RNA even without the canonical RNA-binding site (RBS) domain. Furthermore, the authors show that disrupting these motifs affects the protein's oligomerization dynamics, recruitment of endogenous GEMIN5, ribosome binding, and translation regulation.

Overall, this is an excellent work that provides key features of GEMIN5 biology.
The authors should address the following issues to improve the quality of their manuscript.

1. The manuscript contains several unremoved document markups and formatting errors such as leftover track-change codes and stray lines in the margins which diminish the overall presentation quality. The authors should go over the manuscript and correct these errors.
2. The frequent use of short, disconnected statements creates a fragmented flow throughout the Results and Discussion sections, making the complex biochemical findings harder to follow.
3. The authors' heavy reliance on overexpressed tagged constructs in non-neuronal HEK293 cells may introduce non-physiological artifacts regarding both protein oligomerization and RNA-binding affinity. The authors should acknowledge this in the discussion section.
4. The structural interactions between RNA and the disordered regions rely heavily on AlphaFold predictions rather than experimental structural determinations (such as high-resolution Cryo-EM or X-ray crystallography).

Response to reviewers

We thank the reviewers for their thoughtful comments and suggestions that have helped us to improve the quality of our manuscript. We are happy to see that the three reviewers acknowledged the significance of our work. We are providing a point-to-point response to the comments below.

Reviewer #1 (Comments to the Authors (Required)):

The manuscript by Francisco-Velilla, R., et al. presents the biochemical, structural, and functional characterisation of the RNA-binding domain of GEMIN5. The authors identify two key uncharacterized regions that together constitute an extended RNA-binding surface. Using truncation mutants, site-directed mutagenesis, in vitro EMSAs, cellular pulldown assay, and AlphaFold modelling, the study convincingly links the structural features to RNA binding, self-dimerisation, and ribosome association. Overall, this is a well-structured and cohesive study that I recommend for publication after the authors address a few minor points in the text.

Response: We are grateful to the reviewer for his/her positive view of our manuscript, and also for commenting on the importance and significance of our current work. We appreciate his/her kind words on the organization of our manuscript.

Minor points:
Briefly elaborate on the structural rationale for why extending the TPR domain to TPRα20/26 restores RNA binding.

Response: To address this point, we revised the text to better explain the rationale for extending the TPR domain up to TPRα20/26 to include helices 18-26, as follows (p. 4):
"G5C encompasses several helical domains, including the TPR-like (helices 1-17), followed by a MID region, which comprises helices 18-26 (24). Another tract of helices, from 27 to 32, forms part of the RBS domain (22). Computational analysis of disorder propensity defined by flDPnn (39,40) predicted the presence of a flexible loop between helices 19 and 20, characterized by a stretch of four consecutive serine amino acids, and a heavily disordered region between helices 26 and 27. In addition, the IDR region contains a cluster of acidic residues (Fig 1B), immediately downstream of aromatic and basic residues which determine the RNA-binding ability of a short version of the protein, termed RBS1 (23). The potential involvement of each of these flexible regions in regulating RNA binding was not known. Therefore, we set up to explore the possibility that an extended TPR region could also be involved in RNA binding."

Ensure figure legends clearly state sample sizes/replicates for the oligomerisation Western blots (Fig. 4).

Response: As requested, we have added the sample size in the corresponding figure legend (p. 19):
"SDS-resistant oligomer assembly by GEMIN5 fragments. HEK293 cells expressing the Xpress-His constructs (G5C, G5C-Leu, TPRα26, or the TPR alone) in independent triplicate assays were lysed, and stored at -70ºC until used.

Keep the AF modelling explicitly framed as a predictive structural hypothesis throughout.

Response: The requested correction has been done as follows (p. 9):

"More importantly, the G5C-RNA AlphaFold model shows D5 RNA surrounded by residues R_{1035} and K_{1061} within the TPR-like module of one subunit and the $S_{1141-1144}$ motif of the other subunit (Fig 5A).
Further supporting the involvement of the ser-rich motif for RNA-binding, AlphaFold modeling of the TPRα26 protein reveals the assembly of an elongated dimer engulfing the RNA via residues R_{1090}, K_{1061}, and R_{1035} of the TPR-like moiety (Fig 5B). The RNA-TPRα26 elongated dimer is fully conserved on the model of the RNA-G5C structure (Fig 5C)".

Reviewer #2 (Comments to the Authors (Required)):

GEMIN5, as the RNA-binding protein of the SMN complex linked to human disease, comprises an N-terminal WD40 repeat domain and a C-terminal region (G5C) that includes a tetratricopeptide repeat (TPR)-like dimerization module, a non-canonical RNA-binding site (RBS), and an intrinsically disordered region (IDR), but the potential role of the IDR remains obscure. In this manuscript, Rosario et al. found the RNA-binding domain of GEMIN5 consists of an extended binding surface comprising a serine-rich loop and an acidic cluster within the IDR and the specific modules within the C-terminal helical region modulate RNA-binding, recruitment of the endogenous protein, ribosome binding. Overall, these findings are potentially interesting and most of the experiments are well done and support their conclusions. Following comments may help for further improvement.

Response: We thank the reviewer for raising these concerns.

1.In Figure 1A, the start and end amino acid residues of all truncated constructs should be mapped according to the full-length protein sequence.

Response: We thank the reviewer for this suggestion. As requested, we have added the amino acid residues of all truncated proteins in the legend to Figure 1A showing. This information is also given in Material and Methods. In our opinion the figure was already too overpacked to add more details.

2.In Figure 1E, RNA-binding in vitro of TPRα28 and G5C full-length protein should be added.

Response: We thank the reviewer for this suggestion. The RNA-binding *in vitro* of the G5C protein was already published in 2022 (reference # 24), as indicated in the current manuscript (p. 5). The RNA-binding of TPRα28 has been analyzed, as shown in the figure below, and it binds RNA, although with a lower affinity than TPRα26. We speculate that this could be due to the deficient structure of the most C-terminal region, although we do not have information about this structural reorganization. Thus, in our opinion this data is not adding significant information in the current manuscript.

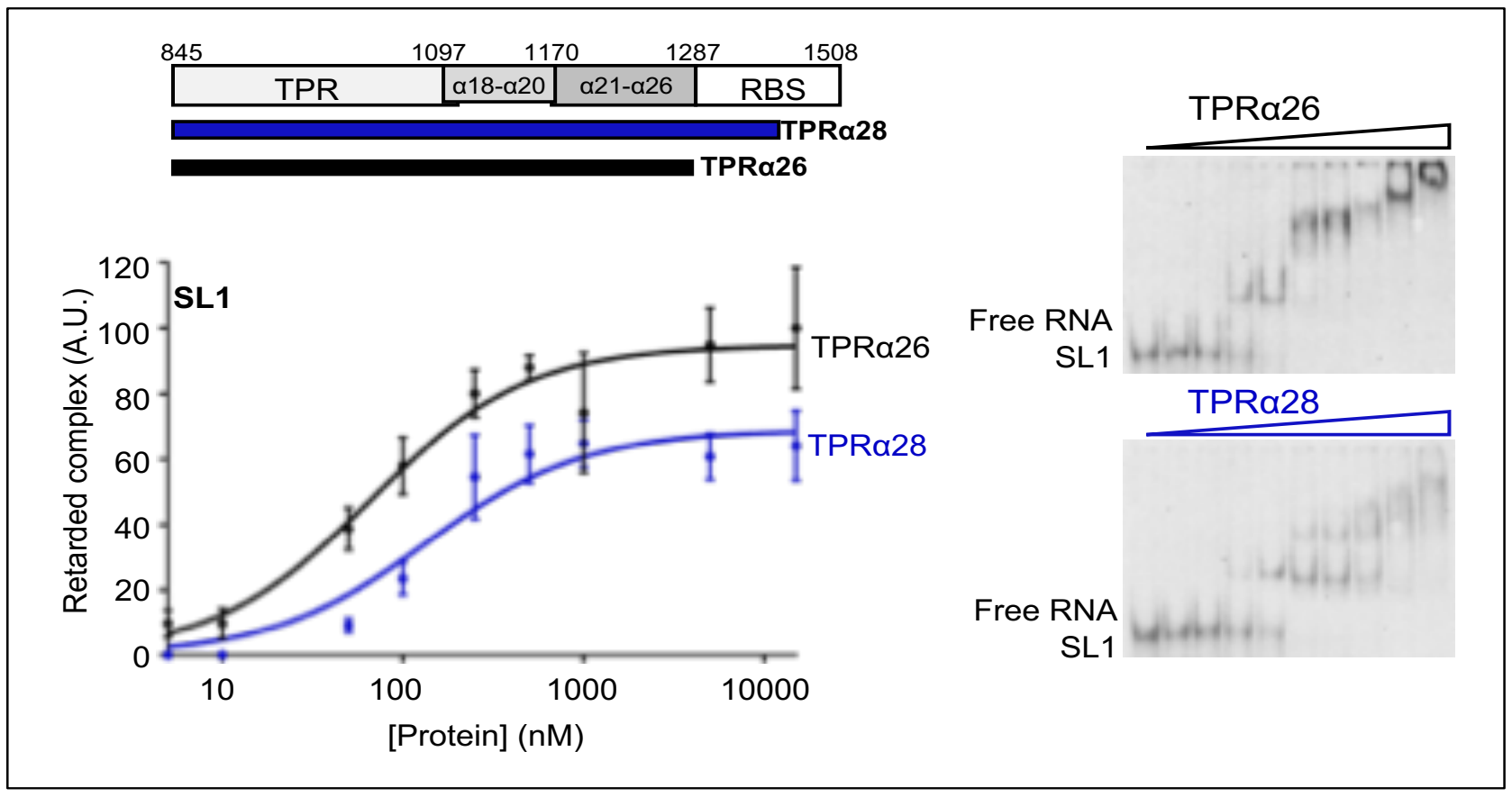

3.All data pertaining to RNA-binding capacity should include calculated Kd values and statistical significance analyses of Kd to demonstrate the differences between the wild-type and mutant proteins.

Response: To address this concern, we have added the Kd values ± SEM in revised Figures 2 and 3.

Reviewer #3 (Comments to the Authors (Required)):

The manuscript by Francisco-Velilla and colleagues investigate the mechanisms governing RNA binding in GEMIN5, an essential RNA-binding protein (RBP) linked to neurodevelopmental disorders. Using a combination of biochemical assays, cell culture truncations/mutations, and computational modeling, the authors identify an extended RNA-binding surface located in GEMIN5's C-terminal domain (G5C). Specifically, they demonstrate that RNA binding is regulated by two distinct unstructured regions: a serine-rich loop within an interhelical region and an acidic cluster within an intrinsically disordered region. The study reveals that while mutations in either region impair RNA binding, the TPR-extended protein moiety remains sufficient to bind RNA even without the canonical RNA-binding site (RBS) domain. Furthermore, the authors show that disrupting these motifs affects the protein's oligomerization dynamics, recruitment of endogenous GEMIN5, ribosome binding, and translation regulation. Overall, this is an excellent work that provides key features of GEMIN5 biology.

Response: We are grateful to the reviewer for his/her positive view of our manuscript, and also for commenting on the importance and significance of our current work.

The authors should address the following issues to improve the quality of their manuscript.
1. The manuscript contains several unremoved document markups and formatting errors such as leftover track-change codes and stray lines in the margins which diminish the overall presentation quality. The authors should go over the manuscript and correct these errors.

Response: Thanks for the comments. The manuscript has been revised to eliminate track-change codes and stray, which were undetected in our own device.

2. The frequent use of short, disconnected statements creates a fragmented flow throughout the Results and Discussion sections, making the complex biochemical findings harder to follow.

Response: The text has been revised to facilitate reading.

3. The authors' heavy reliance on overexpressed tagged constructs in non-neuronal HEK293 cells may introduce non-physiological artifacts regarding both protein oligomerization and
RNA-binding affinity. The authors should acknowledge this in the discussion section.

Response: The manuscript has been revised to indicate the presence of tags in the expressed protein as follows (p. 8):
“The oligomerization property observed in the Xpress-tagged half C-terminal G5C and the protein extended up to helix 26 is associated with the capability to self-dimerize, but also with the ability to recruit the endogenous protein in the HEK293 cellular cytoplasm forming hetero-oligomers (26). This feature, observed in non-neuronal cell extracts, is independent of the tags

added, as the oligomerization of the protein has been observed in different experimental conditions (24,48). Further supporting the intrinsic oligomerization feature of GEMIN5, previous studies reported that the purified GEMIN5 elutes as high molecular weight polymers in gel filtration analysis, with the apparent mobility of a tetramer (48)."

4. The structural interactions between RNA and the disordered regions rely heavily on AlphaFold predictions rather than experimental structural determinations (such as high-resolution Cryo-EM or X-ray crystallography).

Response: Thanks for this comment. This point is clearly indicated in the Discussion (p. 9): "More importantly, the G5C-RNA AlphaFold model shows D5 RNA surrounded by residues R_{1035} and K_{1061} within the TPR-like module of one subunit and the $S_{1141-1144}$ motif of the other subunit (Fig 5A).
Further supporting the involvement of the ser-rich motif for RNA-binding, AlphaFold modeling of the TPRα26 protein reveals the assembly of an elongated dimer engulfing the RNA via residues R_{1090}, K_{1061}, and R_{1035} of the TPR-like moiety (Fig 5B). The RNA-TPRα26 elongated dimer is fully conserved on the model of the RNA-G5C structure (Fig 5C)".

September 29, 2026

RE: Life Science Alliance Manuscript #LSA-2026-03860R

Prof. Encarnacion Martinez-Salas
Centro de Biologia Molecular Severo Ochoa, CSIC-UAM
Genome Dynamics and Function
Nicolas Cabrera, 1, Cantoblanco
Madrid 28049
Spain

Dear Dr. Martinez-Salas,

Thank you for submitting your revised manuscript entitled "Contribution of disordered regions of the essential protein GEMIN5 to RNA binding". Given the strong support already expressed by the reviewers, we have evaluated this revision at the editorial level. We appreciate the changes made to resolve reviewer concerns, however the following points remain outstanding:

Reviewers 1 and 3 both requested that structural claims made with AlphaFold modeling should be softened (a "predictive structural hypothesis" per Reviewer 1). The change made to lines 327-332 does not address this request.

In response to Reviewer 2 point 2, you noted that RNA binding of the G5C construct has been shown in your prior publication. The reference to this publication (lines 171-172) should refer readers to the RNA binding data in that publication, per the request made by this reviewer.

Reviewer 3 requested that limitations inherent to this work should be acknowledged given reliance on overexpressed tagged constructs. The support from prior publications, noted on lines 290-291, does not address this request.

We would be happy to publish your paper in Life Science Alliance pending resolution of the above issues, and final revisions necessary to meet our formatting guidelines.

MANUSCRIPT ORGANIZATION AND FORMATTING:

To avoid unnecessary delays in the acceptance and publication of your paper, please read the following information carefully. Full guidelines are available on our Instructions for Authors page, https://www.life-science-alliance.org/authors

Submission of a paper that does not conform to Life Science Alliance guidelines will delay the acceptance of your manuscript.

-Please upload all figure files as individual files, including the supplementary figure files; all figure legends should only appear in the main manuscript file.
-Please upload your Tables in editable .doc or Excel format.
-Please add the X and Bluesky handles of your host institute/organization, as well as your own and/or one of the authors, in our system.
-Please add your main, supplementary figure, and table legends to the main manuscript text after the references section.
-Please indicate the antibody concentrations used for immunodetection in the methods section.
-We noted that the gel-shift blot for TPRa26 shown in Figure S1 is identical to that shown in Figure 1E. This reuse is acceptable but the duplication must be noted in the legend for Figure S1.
-We noted that blots for wt TPRa26 in Figure 4A (right panel) and Figure 4B (wt lanes) are identical. We suggest adjusting this figure to eliminate the duplicated blots and wording the figure legend as needed to describe the controls used in these assays.

We encourage our authors to provide original source data, particularly uncropped/-processed electrophoretic blots and spreadsheets for the main figures of the manuscript. If you would like to add source data, we would welcome one PDF/Excel-file per figure for this information. These files will be linked as supplementary "Source Data" files.

It is Life Science Alliance policy that if requested, original data images must be made available to the editors. Failure to provide original images upon request will result in unavoidable delays in publication. Please ensure that you have access to all original data images prior to final submission.

If you are planning a press release on your work, please inform us immediately to allow informing our production team and scheduling a release date.

We welcome submissions of potential cover images for the issue of LSA in which your work would appear. If you have high quality images associated with this work, please feel free to email these, with a caption, to the journal office.

LSA encourages authors to provide a 30-60 second video where the study is briefly explained. These videos will be appear embedded with the manuscript online at Life Science Alliance, and on social media to promote the published paper and authors (for examples, see https://docs.google.com/document/d/1-UWCfbE4pGcDdcgzcmiuJl2XMBJnxKYeqRvLLrLSo8s/edit?usp=sharing). Corresponding or first-authors are welcome to submit the video. Please submit only one video per manuscript. The video can be emailed to contact@life-science-alliance.org

FINAL FILES:

To upload the final version of your manuscript, please log in to your account: https://lsa.msubmit.net/cgi-bin/main.plex
You will be guided to complete the submission of your revised manuscript and to fill in all necessary information. Please get in touch in case you do not know or remember your login name.

The following items are required for acceptance.

-- An editable version of the final text (.DOC or .DOCX) is needed for copyediting (no PDFs).

-- High-resolution figure, supplementary figure and video files uploaded as individual files: See our detailed guidelines for preparing your production-ready images, https://www.life-science-alliance.org/authors

-- Summary blurb (enter in submission system): A short text summarizing in a single sentence the study (max. 200 characters including spaces). This text is used in conjunction with the titles of papers, hence should be informative and complementary to the title. It should describe the context and significance of the findings for a general readership; it should be written in the present tense and refer to the work in the third person. Author names should not be mentioned.

The license to publish form must be signed before your manuscript can be sent to production. A link to the license to publish form will be available to the corresponding author only. Please take a moment to check your funder requirements.

Reviews, decision letters, and point-by-point responses associated with peer-review at Life Science Alliance will be published, alongside the manuscript. If you do want to opt out of having the reviewer reports and your point-by-point responses displayed, please let us know immediately.

Thank you for your attention to these final processing requirements. Please revise and format the manuscript and upload materials as soon as you are able.

Thank you for this interesting contribution to the literature. We look forward to publishing your paper in Life Science Alliance.

Sincerely,

Tim Fessenden
Scientific Editor
Life Science Alliance

October 7, 2026

RE: Life Science Alliance Manuscript #LSA-2026-03860RR

Prof. Encarnacion Martinez-Salas
Centro de Biologia Molecular Severo Ochoa, CSIC-UAM
Genome Dynamics and Function
Nicolas Cabrera, 1, Cantoblanco
Madrid 28049
Spain

Dear Dr. Martinez-Salas,

Thank you for submitting your Research Article entitled "Contribution of disordered regions of the essential protein GEMIN5 to RNA binding". It is a pleasure to let you know that your manuscript is now accepted for publication in Life Science Alliance. Congratulations on this interesting work.

The final published version of your manuscript will be deposited by us to PubMed Central upon publication.

Your manuscript will now progress through copyediting and proofing. It is journal policy that authors provide original data upon request.

Your article will publish open access upon publication under a CC-BY license.

Reviews, decision letters, and point-by-point responses associated with peer-review at Life Science Alliance will be published, alongside the manuscript. If you do want to opt out of having the reviewer reports and your point-by-point responses displayed, please let us know immediately.

IMPORTANT: If you will be unreachable at any time, please provide us with the email address of an alternate author. Failure to respond to routine queries may lead to unavoidable delays in publication.

Scheduling details will be available from our production department. You will receive proofs shortly before the publication date. Only essential corrections can be made at the proof stage so if there are any minor final changes you wish to make to the manuscript, please let the journal office know now.

DISTRIBUTION OF MATERIALS:
Authors are required to distribute freely any materials used in experiments published in Life Science Alliance. Authors are encouraged to deposit materials used in their studies to the appropriate repositories for distribution to researchers.

You can contact the journal office with any questions, contact@life-science-alliance.org

Again, congratulations on a very nice paper. I hope you found the review process to be constructive and are pleased with how the manuscript was handled editorially. We look forward to future exciting submissions from your lab.

Sincerely,

Tim Fessenden
Scientific Editor
Life Science Alliance
