# Supplementary material for: Contribution of disordered regions of the essential protein GEMIN5 to RNA binding
Source: Life Sci Alliance. 2026 Oct 27;10(1):e202603860. doi: 10.26508/lsa.202603860 (PMC13714362; doi:10.26508/lsa.202603860)
Supplement: Supplementary file 7 [file LSA-2026-03860_TableS1.docx]

**Supplementary Table 1**

| **CONSTRUCT** | **PRIMER** | **SEQUENCE** |
| --- | --- | --- |
| pET-TPRα20 | BamHI_G5_845-1286 s | atggatccgctcgttccttgctt |
|  | NotI_G5_1170 as | atgcggccgctcaaaggctgaagatgctc |
| pET-α18-α26 | BamHI_G5_1098 s | atggatccgccaacaactgggtg |
|  | NotI_G5_845-1286 as | atgcggccgctcaatacagacgcccataa |
| pET-TPRα26 EE_1132-1133_AA | E1132A/E1133A s | ctctgaaagctgctttgccgccagatgcctggacag |
|  | E1132A/E1133A as | ctgtccaggcatctggcggcaaagcagctttcagag |
| pET-TPRα26 E_1138_A  pcDNA3-Xpress-TPRα26-E_1138_A | E1138A s | ggaggagcttttgcccgctgaaagctgcttttcc |
|  | E1138A as | ggaaaagcagctttcagcgggcaaaagctcctcc |
| pET-TPRα26 SSS_1141-1143_AAA  pcDNA3-Xpress-TPRα26-SSS_1141-1143_AAA | S1141A/S1142A/S1143A s | ccaagtgtggtaagaggcggcggctttgccctctgaaagctgctt |
|  | S1141A/S1142A/S1143A as | aagcagctttcagagggcaaagccgccgcctcttaccacacttgg |
| pET-TPRα26 SY_1144-1145_AA  pcDNA3-Xpress-TPRα26-SY_1144-1145_AA | S1144A/Y1145A s | ccgtgttccaagtgtgggcagcggaggagcttttgccct |
|  | S1144A/Y1145A as | agggcaaaagctcctccgctgcccacacttggaacacgg |
| pET-TPRα26 HW_1146-1148_AA | H1146A/W1148A s | ccttcggtgcccgtgttcgcagtggcgtaagaggaggagctttt |
|  | H1146A/W1148A as | aaaagctcctcctcttacgccactgcgaacacgggcaccgaagg |
| pET-TPRα26 E_1153_A | E1153A s | cacgaaaggccctgcggtgcccgtgtt |
|  | E1153A as | aacacgggcaccgcagggcctttcgtg |
| pcDNA3-Xpress-TPRα20 | TPR/a18-a20 mut TAA s | actgctcaggggtttaaaggctgaagatgctcttccaca |
|  | TPR/a18-a20 mut TAA as | tgtggaagagcatcttcagcctttaaacccctgagcagt |
| pET-RBS1 EE_1324-1325_AA | E1324A/E1325A s | agtttcagggtccgttgctgcagtgctggcagtgtc |
|  | E1324A/E1325A as | gacactgccagcactgcagcaacggaccctgaaact |
| pET-RBS1 EE_1347-1348_AA | E1347A/E1348A s | ctcagcattcgctcacctgctgctgtgagtctcaagtcta |
|  | E1347A/E1348A as | tagacttgagactcacagcagcaggtgagcgaatgctgag |
| pET-RBS1 D_1319_A | D1319A s | tcagtgctggcagtggctaactgctggcttg |
|  | D1319A as | caagccagcagttagccactgccagcactga |
| pET-RBS1 S_1322_A | S1322A s | ggtccgtttcttcagtggcggcagtgtctaactgct |
|  | S1322A as | agcagttagacactgccgccactgaagaaacggacc |
| pET-RBS1 T_1326_A | T1326A s | tcagggtccgcttcttcagtgctggcagtg |
|  | T1326A as | cactgccagcactgaagaagcggaccctga |
| pET-RBS1 DE_1327-1329_AA | D1327A/E1329A s | tctggctgagaagttgcaggggccgtttcttcagtgc |
|  | D1327A/E1329A as | gcactgaagaaacggcccctgcaacttctcagccaga |
| pET-RBS1 E_1329_A | E1329A s | ctctggctgagaagttgcagggtccgtttcttc |
|  | E1329A as | gaagaaacggaccctgcaacttctcagccagag |
| pET-RBS1 S_1331_A | S1331A s | tggctctggctgagcagtttcagggtccg |
|  | S1331A as | cggaccctgaaactgctcagccagagcca |
